# *To use or not to use*: Exploring factors influencing the uptake of modern contraceptives in urban informal settlements of Mumbai

**DOI:** 10.1371/journal.pgph.0000634

**Published:** 2023-03-02

**Authors:** Manjula Bahuguna, Sushmita Das, Sushma Shende, Shreya Manjrekar, Shanti Pantvaidya, Armida Fernandez, Anuja Jayaraman

**Affiliations:** SNEHA (Society for Nutrition, Education and Health Action), Mumbai, Maharashtra, India; University at Buffalo, UNITED STATES

## Abstract

Rapid urbanization and a high unmet need for family planning in urban informal settlements point to the significance of identifying gaps that exist in the path of voluntary uptake of contraceptives. We undertook this study to better understand the perspectives related to family planning among women living in informal settlements of Mumbai. We used a mixed-methods approach, including a cross-sectional survey with 1407 married women of reproductive age and face-to-face in-depth interviews with 22 women, both users and non-users of modern contraceptives. 1070 (76%) of the participants were using modern contraceptives and women’s age, education, parity, socioeconomic status and exposure to family planning interventions were the main determinants of contraceptive use. Poor contraceptive awareness before marriage coupled with social norms of early childbearing and completing family resulted in unplanned and less spaced pregnancies even among current users. In such cases, women either continued with the pregnancy or opted for abortion which sometimes could be unsafe. The decision to use contraceptives was taken in most cases after achieving the desired family size and was also influenced by belief in traditional methods, fear of side effects, spousal/family awareness and counselling by frontline workers. We recommend strengthening of sexual and reproductive health component of adolescent health programs. It is pertinent to inform women about their reproductive rights and most importantly empower them to practice these rights. This can be achieved by increasing women’s age at marriage and continued promotion of formal education. Widespread misconceptions related to the side effects of modern methods need to be mitigated via counselling. Referral, follow-up, and suggestions on available choices of contraceptives should be given in case women face any side effects from the use of contraceptives. At the same time, improving spousal awareness and communication regarding family planning will allow couples to make informed decisions. Finally, roping in role models in the community will create an environment conducive to operationalizing rights-based family planning.

## Introduction

Family planning is a cross-sectoral intervention vital to achieving the Sustainable Development Goals (SDGs) and to bringing transformational benefits of good health and well-being to families [1]. In the last few decades, the focus of family planning programs has shifted from a target-based population control approach to a voluntary human rights-based approach [2]. This paradigm shift promotes reproductive rights related to reproductive self-determination, access to reproductive and sexual health information and services and to equality and non-discrimination [3]. Despite the change in approach, data indicates that in 2019, of 1.9 billion women of reproductive age (15–49 years) worldwide, only 842 million (44%) were using modern methods of contraception and about 190 million (10%) wanted to avoid pregnancy but were not using any method [4].

The Family Planning (FP) 2020 initiative, a global partnership to encourage country-level progress on family planning goals also advocates for the rights of the individual to decide the number and timing of children by giving them full information about family planning methods and improving access to contraceptives [5]. India’s commitment to achieving FP 2020 goals have driven the country’s efforts to expand the reach and coverage of its family planning services through measures like integrating family planning with reproductive, maternal, newborn, child and adolescent health strategy, launching new contraceptives, and media campaigns to create awareness [6]. India has made considerable progress in improving the modern contraceptive prevalence rate (CPR) from 36.1% in 1990 to 52.2% in 2015 but heterogeneity across states has also been reported [7]. As per National Family Health Survey-5 (2019–2020), Maharashtra, one of the most urbanized states in India, has not shown much improvement in both CPR (62.6% to 63.8%) and unmet need for family planning (9.7% to 9.6%) from the previous survey which was conducted in 2015–2016 [8].

Similar to other countries in the world, India is witnessing a rapid pace of urbanization [9, 10] but the haphazard and unplanned nature of urbanization in the country has led to an increase in the population residing in informal settlements [11, 12]. In Mumbai, the capital city of Maharashtra nearly 42% of the residents live in slums [12]. People living in these informal settlements are extremely vulnerable to diseases and injuries due to poor living conditions and lack of access to health services [13, 14]. Women particularly are susceptible to sexually transmitted and reproductive tract infections [15, 16]. They practice less family planning in comparison to their urban non-slum counterparts [17, 18] and also have a high unmet need for family planning [19–22]. Mere availability does not ensure the timely and appropriate use of contraceptives, several aspects solely or in combination determine the use or non-use of contraceptives by women. Some quantitative studies from similar settings have highlighted the importance of women’s age, education, duration of the marriage, number of pregnancies, occupation, husband’s education, socio-economic status and exposure to health systems in the uptake of family planning services [19, 21, 23–26]. There have been only a few qualitative studies that have explored family planning practices and the acceptance of modern contraceptives in India [27–30]. We could not find any study looking at the viewpoint of both users and non-users of contraceptives in urban informal settlements in India.

Considering the expected higher pace of urbanization in lower- and middle-income countries in coming decades [9], it is crucial that we understand the family planning needs and the reasons for both use and non-use of contraceptives among women residing in urban areas, particularly informal settlements. Also, it is indispensable to comprehend any gaps or barriers that limit access to voluntary family planning services to achieve the 2030 agenda for sustainable development.

In this study, we have attempted to achieve an in-depth understanding of family planning and the use of modern contraceptives among women residing in urban informal settlements of Mumbai. We believe that findings from this study could provide inputs to develop strategies to improve voluntary uptake of family planning services in similar settings. Given the context, this mixed-methods study aims 1) to examine women’s perspectives related to family planning and modern contraceptives and 2) to identify factors associated with the use or non-use of modern contraceptives in urban informal settlements of Mumbai.

## Methods

This study was conducted in Malvani, an informal settlement of Mumbai in the state of Maharashtra. For administrative purposes, Mumbai has 24 municipal wards across three zones; city, central and western. Malvani is one of the most populated slum pockets of the P-North municipal ward in the western suburbs of Mumbai [31]. Of all 24 municipal wards of Mumbai, P-North with a huge migrant population, insecure livelihood, illegal housing, poor education, and health facilities ranks nineteenth in Human Development Index [32].

This study was based in the project area of a Non-Government Organization (NGO), the Society for Nutrition, Education and Health Action (SNEHA) covering an estimated population of 60,000. SNEHA has been working in Malvani since 2015 on issues related to reproductive, maternal and child health and nutrition. The project used an integrated life cycle approach to work with married women of reproductive age (15–49 years) and children aged 0–6 years to achieve improvement in health and nutrition outcomes. Family planning intervention was a key component of the project wherein intervention activities included regular home visits to provide information on family planning methods, particularly modern contraceptives, counselling to negate myths and misconceptions regarding the use of modern contraceptives, collaborating with public health systems for service provision in terms of distributing condoms and pills, appropriate referrals and helping women in reaching hospitals for uptake of contraceptives.

As a part of the project, we conducted an in-depth inquiry to understand women’s beliefs, perceptions and factors which influence their decisions to use or not to use modern contraceptives. We used project’s endline data for quantitative analysis which allowed us to have a better understanding of factors that influenced the women’s decision to use contraceptives [33]. Further, interviews with both users and non-users of contraceptives allowed us to do a comparative analysis of the data to get some valuable insights into the family planning needs of the women in these vulnerable communities. Findings were used to strengthen the project’s implementation strategies related to family planning intervention.

### Qualitative methods

Qualitative data was collected between August and December 2017. We used the project’s routine data to recruit participants for the study. We defined a woman as a “user” if she or her partner was using any modern method of contraception at the time of interview and we defined a woman as a “non-user” if she or her partner was not using any modern method of contraception at the time of interview. After generating the initial list, we went to the project site to confirm the current status of contraceptive use. Before enrolling participants for interviews, they were asked, “Which method of family planning are you or your partner using?” and then categorized women as a user or non-user. Women who were using traditional methods such as withdrawal and safe-period were considered non-user. We purposely selected married women aged 30 years or less for interviews to get insights into the process of decision-making related to contraceptive use after marriage. We also took help of the project’s frontline workers in identifying a diverse set of participants including those who were reluctant to use contraceptives, had discontinued the use of contraceptives, or had become a user recently. This helped us gather rich data to understand the women’s perspective and to comprehend possible strategies to address the existing barriers to contraceptive use. We took prior appointments with all the participants and made sure that they were alone at the time of interview owing to the sensitivity of the topic. Based on the participant’s wish and convenience, interviews were conducted either at the participant’s house or at the project office. During data collection, we used the notion of information redundancy to identify the saturation in the data and stopped recruiting more participants after an in-depth exploration of the women’s perception of modern contraceptives and the factors which influenced their uptake of modern methods of family planning. During data analysis, we reconfirmed saturation based on the emerging themes across cases.

We used a guide to help conduct our interviews. The guide explored topics related to the perception of family planning and modern contraceptives in addition to reasons which motivate or challenge the use of modern contraceptives among users and non-users. We also collected some retrospective data to understand the process of becoming a user or non-user after marriage. The interview guide was prepared in English and translated to Hindi. Interviews were conducted by author MB along with two assistants who helped in recording and taking notes during the interview. Interviews were conducted in Hindi and were voice-recorded. Qualitative data from interviews was translated and transcribed verbatim into English and pseudonymized. After going through the initial few transcripts, we affixed the preliminary codes and developed a coding index. Data was collated into groups identified by codes which allowed us to assess the main points that reappeared throughout the data. Patterns among codes were identified and were sorted to form main themes. We revisited data to ensure an accurate representation of data by the finalized themes.

### Quantitative methods

We used data from the project’s endline survey conducted between January and March 2020 to assess the contraception prevalence and its determinants in informal settlements of Mumbai. The inclusion criteria for the survey was married women of reproductive age (15–49 years). Sample size requirements were based on differences in the prevalence of wasting in baseline and end line. Assuming a detectable difference of 5% in wasting in children aged 0–6 years, at 85% power with two-tailed test, a 5% margin of error and 5% non-response rate for refusals and erroneous data which is based on earlier surveys in the area, the final sample size required was 1553. The sampling universe was 14,000 households across 73 clusters of approximately 200 households each. A stratified random sampling method was used for the selection of the sample. Predefined boundaries of the clusters were used as the sampling frame. All households with children aged 0–6 years were listed for each cluster separately and a random sample from each list was drawn to achieve the required sample size.

The list of randomly selected households was given to a well-trained team of field investigators for conducting the survey. The questionnaire included questions on demographic and socio-economic information, maternal and reproductive health (parity, birth history, antenatal, delivery and postnatal care, family planning and contraceptives), child health (feeding habits, immunization, morbidity and nutritional status) and uptake of health services from government and private providers including non-governmental organizations (NGOs).

For analysis, we calculated Contraceptive Prevalence Rate (CPR) by assessing the percentage of married women of reproductive age (15–49 years) who were using or whose partners were using any modern contraceptive method at the time of survey out of eligible married women of reproductive age (15–49 years) in the sample. Factors associated with contraceptive use among married women of reproductive age were explored using logistic regression models. As a dependent variable, we categorized the use of modern contraceptives as “1” and the non-use of modern contraceptives as “0”. Independent variables were chosen based on literature related to the determinants of use of modern contraceptives and feedback of the project team. The final model included women’s age, education, employment status, religion, number of children, duration of residence in Mumbai, husband’s education, uptake of family planning services from an NGO or public health system, number of household members and asset index quartile as independent variables. Respondents were considered employed if engaged in either formal or informal activity. We collected data on household assets and developed an index of household wealth by assigning standardized weights to the first component of the principal component analysis of the asset data. We divided the resultant factor into quartiles to describe the socio-economic position of our study participants [34]. For each explanatory variable, the crude odds ratio was presented along with the adjusted odds ratio and 95% confidence intervals (CI). All statistical analysis was conducted in STATA 12.0 (StataCorp, College Station, TX).

#### Ethical considerations

Ethical approval for the study was obtained from the Institutional Ethics Committee, the Bandra Holy Family Hospital & Medical Research Centre, Mumbai. We explained the purpose of the study to all participants in detail. All respondents gave their written informed consent before participation in qualitative interviews and quantitative survey. The study did not include any minors.

## Results

### Qualitative findings

We interviewed 22 women of which 13 were users of modern contraceptives and 9 were non-users of modern contraceptives (Table 1).

**Table 1:** Demographic characteristics of the interview participant from the project area in Malvani, Mumbai (N = 22).

**Current contraceptive status**	
User	13
Non-user	9
**Age**	
< = 20 years	1
21 to 25 years	7
26 to 30 years	14
**Education**	
No formal schooling	7
Less than 6 years of schooling	0
6–10 years of schooling	13
Higher than 10 years of schooling	2
**Religion**	
Muslim	17
Hindu	5
**Employment status**	
No work	16
Informal work	6
**Number of children**	
1 to 2	12
3 or more	10
**Age at marriage**	
<18 years	9
> = 18 years	13

The qualitative findings were categorized under three broad themes. The first theme was about awareness of family planning and different methods of contraception among both users and non-users. The second theme tried to capture the underlying beliefs of women related to modern and traditional methods of family planning which influenced their decision to use or not to use modern contraceptives. The third theme explored barriers and enablers influencing the uptake of modern contraceptives by women (Table 2).

**Table 2:** Summary of themes and sub-themes.

Themes	Sub-themes
Perception about family planning	Importance of family planning
	Awareness of modern and traditional family planning methods
Beliefs shaping the decision to use or not use modern contraceptives	Trust in traditional methods to achieve the benefits of family planning and reluctance to use modern contraceptives
	Experiences of unplanned pregnancies and abortions
	Desire to use modern contraceptives
Barriers and enablers influencing the uptake of modern contraceptives by women	Fear of side effects
	Spousal awareness and communication
	Role models in the family
	Counselling and moral support by frontline workers
	Societal norms, cultural and religious beliefs

### Perception about family planning

All the participants were well aware of modern contraceptives. When asked about their views regarding family planning, both users and non-users shared that it helps in shaping a better future for their children and families. They reported the cost of children’s education and upbringing as one of the main reasons for realizing the importance of family planning.

*“There is a price rise in everything including education; it is a big problem*. *If we have more children*, *we won’t be able to give them a good upbringing*. *Sending them to good schools*, *giving them good food and clothes; we won’t be able to do anything if there are more children*. *That is why I don’t want more children*.” ***(User*, *26 years*, *3 children*, *married at the age of 18 years*, *first pregnancy at the age of 21 years***)

*“It’s troublesome to have a lot of kids*. *We are responsible to provide better care*, *quality education and a good life to our kids*. *We need to be a small family*, *as having a lot of kids will not allow us to have a proper standard of living*. *We may not be able to focus on all the children*. *We will not be able to provide quality education; possibly few of them (children) might not even get the education*.*”* ***(Non-user*, *22 years*, *2 children*, *married at the age of 17 years*, *first pregnancy at the age of 19 years***)

All respondents including non-users knew about different types of modern contraceptives. They mentioned various methods like condoms, pills, Copper-T (intrauterine device), *Antara* (injectable) and female sterilization. Female sterilization was mostly referred to as ‘operation’ in the community.

### Beliefs shaping the decision to use or not use modern contraceptives

Among our study participants, we found that the decision to use or not use modern contraceptives originated from the belief in modern methods to achieve the small and well-spaced family. Users and non-users shared their views regarding this. Non-users desired the benefits of family planning but not all of them believed in modern contraceptives to achieve these benefits. Therefore, non-users could be divided into two categories; 1) those who did not believe in modern contraceptives and 2) those who believed in modern contraceptives. According to the non-users in the first group, the benefits of family planning such as limiting the number of children and spacing between them could be achieved by using traditional methods such as withdrawal and calendar. They shared that these methods were better and hassle-free as they only required mutual understanding between husband and wife. When asked about the risks of unwanted pregnancies, women shared that the chances of getting pregnant were less likely as they practiced self-control and knew their limitations. Women also talked about the trust they had in their partners when it came to the practice of traditional family planning methods.

*“We don’t use any method*, *because he (the husband) understands it and knows it all*. *He is educated in Mumbai*. *He says we should not use it (contraceptives) and harm our health*. *10–15 days he stays out but he doesn’t use anything*.*”* ***(Non-user*, *21 years*, *1 child*, *married at the age of 19 years*, *first pregnancy at the age of 20 years***)

“*It’s the same*, *I can control now*, *similarly I will control forever*. *I just need to control for a few more years*. *After a certain age*, *periods stop and then I will not get pregnant*. *Simple*, *I just need to be careful for the next 8–10 years*… *That’s it*.*”* ***(Non-user*, *22 years*, *2 children*, *married at the age of 17 years*, *first pregnancy at the age of 19 years***)

Non-users in the second category believed that modern contraceptives were beneficial and better in comparison to traditional methods to achieve the benefits of family planning. They could not use it due to reasons like fear of side effects, and no support from their husband and their families. During our interaction with this set of non-users, we sensed their fear of unintended pregnancies and their intense desire to avoid them. They shared their experience of such pregnancies and their attempts to abort it first through home remedies and later by medicines.

*“When I was pregnant*, *I used some red coloured thing with water*. *But nothing happened*. *I even ate food that generates heat in our body but nothing happened to my baby*.*”* ***(Non-user*, *21 years*, *1 child*, *married at the age of 19 years*, *first pregnancy at the age of 19 years)***

*“My daughter was very small when I conceived again*. *So*, *I aborted it*. *I took a tablet from the pharmacy; we had consulted a doctor who had suggested that medicine*.*”* ***(Non-user*, *20 years*, *2 children*, *1 abortion and 1 miscarriage*, *married at the age of 15 years*, *first pregnancy at the age of 16 years***)

Three users in our study also shared similar experiences of abortion which prompted them to use modern contraceptives.

*“When I conceived for the third time*, *I didn’t want to deliver the baby*. *Some said a baby can be aborted if you jump*, *eat papaya*, *fish or spices; it’s all heaty food*. *I did eat papaya*, *but nothing happened*. *I even jumped from the bed*, *but nothing happened*. *Then*, *I had to take medicine*.*”* ***(User*, *28 years*, *4 children and 1 abortion*, *married at the age of 17 years*, *first pregnancy at the age of 18 years***)

*“I told the doctor*, *that I need to undergo an abortion as I have a small kid of four months*. *She said okay*, *I will do an abortion but what will you use after that*. *I said I will use copper-T*. *Then she did an abortion and put copper-T*.*”* ***(User*, *30 years*, *3 children and 2 abortions*, *married at the age of 17 years*, *first pregnancy at the age of 19 years***)

In the case of most of the users, their decision to use contraceptives had emanated only after they had experienced unintended or unplanned pregnancies. It changed their perspective about contraceptives and its value in their lives. These users shared that those unintended pregnancies could have been avoided with the use of contraceptives. (See Box 1 for an illustrative case).

**Box 1. Case of a single mother of four children who used contraceptives after experiencing unintended pregnancies.**

This 28 years old woman lived near her mother-in-law. She got married at the age of seventeen, conceived immediately and delivered her first child just after one year of marriage. She was aware of modern contraceptives but was not sure about using them. She was also scared of the side effects. She had a bad relationship with her husband, as he was unfaithful to her. In the next 10 years, she conceived four more times resulting in three live births and one induced abortion. After the birth of her fourth child, she opted for sterilization. Her husband abandoned her and her children. She felt that she was naive and would have benefitted if she had used contraceptives earlier.

*“I was scared of using contraceptives*. *Few say that it (Copper-T) moves up*, *that’s the reason I didn’t use it*. *Medicines were also available but I never took any and some said that it gives trouble*. *But then it was all uneducated talk*. *We had never used it so no one had any knowledge*.”

*“Now…I feel free*, *I should have done this after my second child*. *I could have been more relaxed if I had not given birth to two more children*.”

Four users in our study said that they always wanted to use contraceptives for good health, planned pregnancies, limiting the number of children and the desired spacing between pregnancies. So, they used contraceptives immediately after marriage and continued to do so after and between pregnancies.

*“I decided that I will need some gap between pregnancies*. *I need to be comfortable and confident that I can take care of the second child*. *I used Copper-T to have that gap*. *I conceived my second baby only when I was comfortable*.*”* ***(User*, *24 years*, *2 children*, *married at the age of 17 years*, *first pregnancy at the age of 18 years***)

In summary, not all women preferred modern methods to limit and maintain spacing between pregnancies. Only a few women used contraceptives right after marriage and planned their pregnancies rest did not use them due to various reasons described in the next section. They shared experiences of unplanned pregnancies which in the case of some current users had acted as a trigger to use modern methods.

### Barriers and enablers influencing the uptake of modern contraceptives by women

Our findings suggest that women’s decision to use contraceptives may not necessarily lead to the actual use of contraceptives. There are multiple factors that influence the uptake of contraceptives. The interplay of these factors determines whether a woman will be a user or a non-user of contraceptives. Some factors we could identify in our qualitative data are described below:

*Fear of side effects*. Among non-users, we found that the fear of side effects of modern contraceptives was the main deterrent to use. Non-users spoke of their concerns related to side effects, some of which were based on their personal experiences and the rest was the perception built due to widespread misconceptions in the community. Of the nine non-users in our study, three had discontinued the use of modern contraceptives after experiencing side effects but the remaining six had not even tried due to the fear of side effects.

*“I don’t use Copper-T anymore*. *I had heavy bleeding during periods*. *My hands*, *legs and back hurt a lot*. *I was having so many problems*. *That is why I don’t use Copper-T*.*”* ***(Non-user*, *28 years*, *4 children and 1 abortion*, *married at the age of 15 years*, *first pregnancy at the age of 15 years***)

*“I feel scared of using contraceptives*. *It is said that if it (Copper-T) does not suit you it goes up in the body*. *Then something will happen and I will have to take it out*. *That is why I don’t use it*. *Aunty living in front of our house says it goes up*, *gets stuck and gets infected*. *That scared me*, *that is why I have not used it yet*.*” (****Non-user*, *21 years*, *1 child*, *married at the age of 19 years*, *first pregnancy at the age of 20 years***)

In our interaction with users, we found that a few of them had also experienced some side effects after using contraceptives but they did not give up and switched to the other methods after inquiring about the choices available with their healthcare providers or frontline workers.

*“Tai (frontline worker) suggested that I use Copper-T*. *She said that it would be very useful and will make me tension-free for the next ten years*, *but it didn’t work well for me*. *I had continuous stomach pain*, *so I removed it*. *Now I am using Antara (injectable)*. *It’s been two months now*.*”* ***(User*, *30 years*, *2 children*, *married at the age of 12 years*, *started living with her husband after gauna (consummation of marriage) ceremony at the age of 18 years*, *first pregnancy at the age of 19 years***)

*Spousal awareness and communication*. Spousal awareness and communication constituted an important factor that influenced the uptake of modern contraceptives. The husband was the final decision maker and women sought the approval of the husband before the uptake of any family planning method. The financial dependency of women on their husbands also weakened their ability to decide for themselves. Here, spousal awareness regarding contraceptives was important.

Non-user participants in our study shared that they rarely had any conversations with their husbands regarding family planning. Women felt uncomfortable talking about contraceptives with their partners. Another reason for not talking to them was that they did not want their husbands to get worried over such trivial things.

*“Volunteers gave me free samples (condoms)*, *I kept them in the cupboard*. *I was feeling shy*, *so I didn’t even show it (condoms) to my husband*.*”* ***(Non-user*, *20 years*, *2 children*, *1 abortion and 1 miscarriage*, *married at the age of 15 years*, *first pregnancy at the age of 16 years***)

Few non-users reported that whenever they tried talking to their husbands about using contraceptives, they avoided the conversation and did not show any interest. One non-user reported that her husband refused to use condoms saying it is required only when someone has multiple sexual partners.

*“He never told me anything about it (contraceptives)*. *I had a word with him once regarding Copper-T*, *but he did not allow its use saying*, *let it be the way it is*.*”* ***(Non-user*, *21 years*, *1 child*, *married at the age of 19 years*, *first pregnancy at the age of 19 years***)

“*I told him (husband) about condom but he said ‘I don’t need it*. *He said that I am not going to see any other woman*, *so why do I need it*.*”* ***(Non-user*, *29 years*, *1 child*, *married at the age of 26 years*, *first pregnancy at the age of 29 years***)

Four users in our study shared that their husbands were aware of the benefits of modern contraceptives. Husbands initiated the conversation just after their marriage, they discussed family planning and using contraceptives. This helped the couple in both limiting and spacing births.

*“Yes*, *we did talk about it (contraceptives) after marriage*, *he said that you don’t use it as you may have some stomach problem*, *so I will use*.*”* ***(User*, *27 years*, *1 child*, *married at the age of 25 years*, *first pregnancy at the age of 25 years***)

*“Yes*, *(after marriage) we both decided that there should be a gap of at least 6 months*. *I have come from another house and now I have to establish a new home*. *Right thing is that we both first take care of each other*.*… like one gets married and immediately has a child*, *it’s not good*.*”* ***(User*, *27 years*, *2 children*, *married at the age of 18 years*, *first pregnancy at the age of 19 years***)

Among the other nine current users, we found that early pregnancies and less spacing between children made them realize the importance of modern contraceptives. These users reported having conversations with their husbands regarding the desire to use contraception which helped them seek information and adopt a method of their choice.

*“My husband uses this (condom) method*. *He said it himself*, *that we do not need more children now*. *He said that we should use this to stop having more children and I also agreed with him*.*”* ***(User*, *26 years*, *3 children and 1 abortion*, *married at the age of 18 years*, *first pregnancy at the age of 19 years***)

*Role models in the family*. Another factor that influenced the women’s decision regarding the uptake of contraceptives is the other users in the family. They were the main source of information on contraceptives for a woman in these communities. They counselled women about the benefits of modern contraceptives mostly after the birth of their first child. These women were the role models for the users as they shared their knowledge and experience of using contraceptives. It helped women to overcome the shame and anxiety associated with the use of any method of family planning and shaped their beliefs about the benefits of contraceptives. Users shared that their mother/mother-in-law/sister-in-law sought information from frontline workers and shared it with them. They also accompanied women to hospitals for the uptake of modern contraceptives.

*“Yes*, *she (mother-in-law) said that if you use this (Copper-T) you won’t get pregnant*. *When you don’t want a child use it and when you want a child then remove it*.*”* ***(User*, *26 years*, *3 children and 1 miscarriage*, *married at the age of 18 years*, *first pregnancy at the age of 19 years***)

*“After her (first child) my mother suggested Copper- T*. *She knew beforehand*, *she herself has used it*. *She had this knowledge*.*”* ***(User*, *24 years*, *2 children and 1 miscarriage*, *married at the age of 17 years*, *first pregnancy at the age of 18 years***)

In the case of non-users, they did not have any users in the family hence there was no support from family members. In fact, families interfered in the couple’s decision related to family size and did not allow women to use contraceptives.

*“She (mother-in-law) heard and said there is no need to use Copper T*. *I said let me go and use it*, *but she refused and didn’t allow me to go out of the house*. *She said*, *you just have one child so hold on for some time and have one or two more children*.*” (****Non-user*, *21 years*, *1 child*, *married at the age of 19 years*, *first pregnancy at the age of 20 years***)

*Counselling and moral support by frontline workers*. Some respondents shared that they were advised about the use of contraceptives by health professionals. However, not in all cases, advice from professionals resulted in immediate uptake due to widespread misconception and fear of side effects in the community. Few current users said that frontline workers who visited them helped in negating their fears about modern contraceptives.

*“In the hospital*, *when I delivered my first baby*, *they (hospital staff) advised me to use copper-T*. *I thought of using it but then I didn’t use it*.*”* ***(User*, *28 years*, *4 children*, *married at the age of 17 years*, *first pregnancy at the age of 18 years***)

*“I didn’t know anything about this*. *That Didi (frontline worker) explained everything to me*. *After that only this thing (using contraceptives) came up in my mind; now I want to use copper-T*. *I believe it would control pregnancy for 5 years and so*.*”* ***(Non-user*, *20 years*, *2 children*, *1 abortion and 1 miscarriage*, *married at the age of 15 years*, *first pregnancy at the age of 16 years***)

*“Girls (frontline workers) from the organization came and they showed us videos on their tablets*. *They explained that copper-T will not go up in the stomach as it’s in the uterus and there is no space left; it may bend but will not go up in the body*. *They gave us this information quite frankly*. *They suggested that it will be a good option or we can also choose operation*.*”* ***(User*, *29 years*, *3 children and 1 abortion*, *married at the age of 22 years*, *first pregnancy at the age of 22 years***)

Frontline workers provided moral support to the women for the uptake of contraception by giving information, filling out forms and accompanying them to the hospital. In case of any side effects, women trusted them for advice and help.

*“I faced so many problems in going and putting copper-T the first time*. *My mother had to go to the health post (Primary health care unit) several times*. *Next time with them (frontline workers)*, *my work happened easily*.*”* (***User*, *24 years*, *2 children and 1 miscarriage*, *married at the age of 17 years*, *first pregnancy at the age of 18 years)***

*Societal norms*, *cultural and religious beliefs*. Findings suggest that women in the community usually were married young. Nine out of twenty-two women in our qualitative sample got married under 18 years of age. Two women were married between the age of 10–12 years but they were sent to live with their husbands only after *gauna* (a north Indian custom and ceremony linked with the consummation of marriage) which took place when they turned 18 years of age.

*I was young 10 years old when got married*. *We marry at a young age but gauna was done after 8 years*. *I came to my husband after 8 years and conceived after 2 months*. *Now we are ganwar (not educated) people*. *Educated people know things but we didn’t know anything (about family planning)*. *Our mother didn’t teach us*. ***(Non-user*, *28 years*, *3 children 1 abortion*, *married at the age of 10 years*, *started living with her husband after gauna (consummation of marriage) ceremony at the age of 18 years*, *first pregnancy at the age of 18 years***)

At the time of their marriage women had no or incomplete information about family planning. Also, family planning was seen as a taboo subject in the community. It was considered a topic to be discussed after marriage only. Both users and non-users shared that they did not receive much information about it before marriage.

*“It’s natural that after you get married then only you get to know about such things*. *Till we are at our parent’s place and not married*, *no one talks about it and neither is one interested or eager to know about the same*. *It’s only after you get married that you visit hospitals and you get this information*.*”* ***(User*, *29 years*, *3 children and 1 abortion*, *married at the age of 22 years*, *first pregnancy at the age of 22 years***)

“*My mother resides nearby but she doesn’t understand anything about it. Maybe she also feels shy like I do. My sisters are also there but they are too young and they haven’t got married yet.” **(Non-user, 20 years, 2 children and 1 abortion and 1 miscarriage, married at the age of 15 years, first pregnancy at the age of 16 years)***

*“No*, *nothing of these (family planning) talks*. *You know it’s very embarrassing*. *No mother can talk about these things*, *if two women are talking and then someone comes*, *they will stop talking about these things*.*”* ***(User*, *30 years*, *3 children*, *married at the age of 20 years*, *first pregnancy at the age of 21 years***)

Women in the community feared that seeking information about contraceptives or discussing it openly might result in labelling them as “women with bad morals”. One user who had recently come to reside in the community from another state shared her concerns about it.

“*I tell people that I don’t know about it (contraceptives) because then they will wonder how I know about these things***.*” (User*, *27 years*, *1 child*, *married at the age of 25 years*, *first pregnancy at the age of 25 years***)

Women also preferred the covert use of contraceptives. Because of that, we found that intrauterine devices were quite popular in the community in comparison to pills or sterilization.

*“No*, *nobody will allow the operation*. *For Copper-T*, *I can go alone and put it but for operation*, *I cannot go alone*. *Everyone will get to know about it then*.*”* ***(User*, *30 years*, *3 children and 1 abortion*, *married at the age of 17 years*, *first pregnancy at the age of 19 years***)

Both users and non-users shared that families expect a child just after marriage. In most cases, it was the pressure of completing the family which forced women to postpone the use of contraceptives. Women also reported the fear of not being able to get pregnant later if they delayed their pregnancy which also restricted the uptake of contraceptives. Women shared a few instances of their families taking them to the clinics for not conceiving a few months after marriage. Both users and non-users reported continuing with the unwanted pregnancies due to fear of not getting pregnant later. They feared that abortion might lead to some complications and they may not get pregnant again.

*“I always felt that because of my weight*, *I would never become a mother*. *I didn’t want kids immediately after marriage but my mother said*, *“what if you can’t get pregnant later*? *It would be difficult*, *so better you continue with this pregnancy*.*”* ***(Non-user*, *22 years*, *2 children*, *married at the age of 17 years*, *first pregnancy at the age of 19 years***)

*“In my village*, *some of my friends tried to conceive after two to three years of marriage*, *Now*, *it’s been ten years and they still don’t have a child*. *That is why I thought that first I will have one child*, *later I will see*.*”* ***(User*, *27 years*, *1 child*, *married at the age of 25 years*, *first pregnancy at the age of 25 years***)

*“Yes*, *if someone had brought it (abortion medicine) to me*, *I would have taken but if something (complications) would have happened*, *everybody would have shouted at me*. *I was scared*.*”* ***(User*, *23 years*, *2 children*, *married at the age of 19 years*, *first pregnancy at the age of 19 years***)

In the community, childbirth was considered as the will of God. Few Muslim non-users compared using contraceptives to the *gunah* (sin) and shared that one should not interfere with God’s blessings by using any method. However, some Muslim users who were using temporary contraceptives like pills and intrauterine devices said that the methods which do not stop childbirth permanently were acceptable. Its only sterilization which was not allowed by their religion and was considered a *gunah*.

“*God is responsible for whatever happens*. *If I get pregnant it is good and if not then nothing*. *It’s all about almighty Allah; he is the one to give me the blessings*.*”* ***(Non-user*, *22 years*, *2 children*, *married at the age of 17 years*, *first pregnancy at the age of 19 years***)

“*Not all other kinds of operations but this operation (sterilization) is gunah*. *In this*, *you are killing a child that is why it’s a gunah*. *When we will die then all kids you have destroyed or have stopped after the operation will be standing and they will catch us*. *That is why I think that operation should not be done*.*”* ***(Non-user*, *28 years*, *4 children*, *married at the age of 16 years*, *first pregnancy at the age of 18 years***)

One Muslim user who had undergone sterilization shared that any method which prevents unintended pregnancies was better than using nothing. According to her frequent abortions were g*unah*.

*“I have talked to so many people* and I *believe that operation is better than frequent abortions*. *It is killing a living soul and also results in the bad health of women*. *This is more of a “gunah”*. *So*, *when you are certain that you don’t want any further children this operation (sterilization) is better***.*” (User*, *27 years*, *2 children*, *married at the age of 18 years*, *first pregnancy at the age of 19 years***)

### Quantitative findings

A total of 1407 women participated in the survey. More than three fourth, 76% of the respondents were using modern contraceptives at the time of data collection. Table 3 shows that among 1070 users, the proportion of women was the highest in the following categories: women more than 30 years of age (39.6%), with completed 6–10 years of schooling (62.0%), husband with completed 6–10 years of schooling (62.6%), living in Mumbai for more than 10 years (63.8%), with a family of 5 or fewer members (61.2%) and used services of both NGOs and public health system (52.8%).

**Table 3:** Socio-economic and demographic characteristics of married women of reproductive age (15–49 years) in urban informal settlements of Malvani, Mumbai.

	Contraceptive users (N = 1070)	Contraceptive non-users (N = 337)
**Age**		
< = 25 years	273 (25.5%)	107 (31.7%)
26–30 years	373 (34.9%)	95 (28.2%)
>30 years	424 (39.6%)	135 (40.1%)
**Education**		
No formal schooling	167 (15.6%)	55 (16.3%)
Less than 6 years of schooling	38 (3.5%)	10 (2.9%)
6–10 years of schooling	663 (62.0%)	198 (58.8%)
Higher than 10 years of schooling	202 (18.9%)	74 (22%)
**Employment status**		
No	886 (82.8%)	288 (85.5%)
Yes	184 (17.2%)	49 (14.5%)
**Religion**		
Muslim	914 (85.4%)	281 (83.4%)
Hindu	137 (12.8%)	51 (15.1%)
Others	19 (1.8%)	5 (1.5%)
**Number of children**		
0 or < = 2	580 (54.2%)	245 (72.7%)
3 or more	490 (45.8%)	92 (27.3%)
**Duration of residence in Mumbai**		
< = 5 years	213 (19.9%)	78 (23.1%)
6 to 10 years	174 (16.3%)	43 (12.8%)
>10 years	683 (63.8%)	216 (64.1%)
**Husband’s education**		
No formal schooling	163 (15.2%)	55 (16.3%)
Less than 6 years of schooling	49 (4.6%)	10 (2.9%)
6–10 years of schooling	670 (62.6%)	198 (58.8%)
Higher than 10 years of schooling	188 (17.6%)	74 (21.9%)
**Number of household members**		
Less than or equal to 5	655 (61.2%)	221 (65.6%)
More than 5	415 (38.8%)	116 (34.4%)
**Uptake of family planning services**		
No services availed	106 (9.9%)	92 (27.3%)
Only NGOs services availed	399 (37.3%)	204 (60.5%)
Both NGOs and public health system services availed	565 (52.8%)	41(12.2%)
**Wealth index**		
Poorest	270 (25.2%)	88 (26.1%)
Quartile 2	257 (24.0%)	80 (23.7%)
Quartile 3	263 (24.6%)	101 (29.9%)
Least Poor	280 (26.2%)	68 (20.2%)

Table 4 presents factors associated with the use of modern contraceptive methods by married women of reproductive age in our sample population. The table suggests that women in the age group of 26–30 years [AOR 1.58, 95% CI 1.08–2.32], with 6–10 years of schooling [AOR 1.51, 95% CI 1.01–2.26] or higher than 10 years of schooling [AOR 1.66, 95% CI 0.99–2.76] and with three or more children [AOR 2.75, 95% CI 1.91–3.94] had higher odds of using modern contraceptives in comparison to young women with no schooling and with two or fewer children. Women who received family planning services from NGOs [AOR 1.80, 95% CI 1.27–2.57] and both NGOs and the public health system [AOR 14.8, 95% CI 9.39–23.35] had higher odds of using modern contraceptives compared to women who had not availed any family planning services. Women belonging to the least poor category had higher odds [AOR 1.74, 95% CI 1.10–2.75] of using modern contraceptives compared to the women in the poorer category.

**Table 4:** Factors associated with the use of modern contraceptive methods by married women of reproductive age (15–49 years) in urban informal settlements of Malvani, Mumbai.

	Crude odds ratio (95% CI)	Adjusted odds ratio (95% CI)
**Age**		
< = 25 years	1	1
26–30 years	1.53 (1.12, 2.11)**	1.58 (1.08, 2.32)*
>30 years	1.23 (0.91, 1.65)	1.30 (0.86, 1.98)
**Education**		
No formal schooling	1	1
Less than 6 years of schooling	1.59 (0.72, 3.48)	1.36 (0.57, 3.22)
6–10 years of schooling	1.31 (0.94, 1.83)	1.51 (1.01, 2.26)*
Higher than 10 years of schooling	1.01 (0.68, 1.50)	1.66 (0.99, 2.76)*
**Employment status**		
No	1	1
Yes	1.22 (0.86, 1.71)	1.10 (0.74, 1.63)
**Religion**		
Muslim	1	1
Hindu	0.82 (0.58, 1.16)	1.07 (0.72, 1.58)
**Number of children**		
0 or < = 2	1	1
3 or more	2.24 (1.72, 2.94)***	2.75 (1.91, 3.94)***
**Duration of residence in Mumbai**		
< = 5 years	1	1
6 to 10 years	1.48 (0.97, 2.26)	1.07 (0.65, 1.75)
>10 years	1.15 (0.85, 1.56)	0.83 (0.56, 1.22)
**Husband’s education**		
No formal schooling	1	1
Less than 6 years of schooling	1.65 (0.78, 3.48)	1.42 (0.63, 3.24)
6–10 years of schooling	1.14 (0.80, 1.61)	0.99 (0.66, 1.49)
Higher than 10 years of schooling	0.85 (0.57, 1.28)	0.69 (0.41, 1.15)
**Uptake of family planning services**		
No services availed	1	1
Only NGOs services availed	1.69 (1.22, 2.35)***	1.80 (1.27, 2.57)***
Both NGOs and public health system services availed	11.96 (7.83, 18.24)***	14.8 (9.39, 23.35)***
**Number of household members**		
Less or equal to 5	1	1
More than 5	1.20 (0.93, 1.55)	0.79 (0.57, 1.09)
**Wealth index**		
Poorest	1	1
Quartile 2	1.04 (0.73, 1.48)	1.11 (0.74, 1.67)
Quartile 3	0.84 (0.60, 1.18)	0.92 (0.61, 1.38)
Least Poor	1.34 (0.93, 1.91)	1.74 (1.10, 2.75)*

Statistical significance

* p value: ≤0.05

** p value: ≤0.01

*** p value: ≤0.001

Quantitative data showed that women with 3 or more children used more contraceptives which corroborates with our qualitative data suggesting the use of contraceptives mostly for limiting the pregnancies after achieving the desired number of children. Also, women who seek help from NGOs and public health systems used more contraceptives as they had access to information and support in the uptake of contraceptives.

## Discussion

This study contributes to the in-depth understanding of women’s perspectives on family planning and contraceptive use in urban informal settlements of Mumbai. Our findings also presented the factors which influence the uptake of contraceptives. The human rights-based approach to family planning broadly encompasses the individual’s access to the information and resources to freely decide the number, spacing and timing of their children, a decision which is free from any form of discrimination and coercion [35]. This study helps us to identify the gaps that still exist in the path to achieving voluntary and rights-based family planning in our community. Barriers at the individual and societal levels need immediate attention to fulfill the reproductive health needs of the women residing in informal settlements. One of the strengths of our study is the use of both quantitative and qualitative methods along with the interviews with both users and non-users. It allowed us to gain some valuable insights into the family planning decisions of women living in urban informal settlements.

A few limitations of our study were that we used cross-sectional survey data which indicated only associations and not causal linkages. Another limitation is that we did not include unmarried, widowed and divorced women in the sample as the focus of the study was only married women of reproductive age. Also, in qualitative interviews, we did not talk to the husbands whose views regarding contraceptives would have shed more light on its influence on the couple’s decision.

Women in our study were well aware of modern contraceptives. Quantitative data also showed that nearly three-fourths of the participants were using contraceptives. However, qualitative findings highlighted that contraceptive use was considered mostly for limiting the number of children. Only a few users in our study used contraceptives right after their marriage and were able to plan and space their pregnancies. The rest of the users thought of using contraceptives after experiencing the agony of unintended pregnancies and induced abortions. Our quantitative data indicated that women in a higher age group and with a greater number of children were using more contraceptives in comparison to younger women with a fewer number of children, findings similar to other studies [25, 26, 36, 37]. It can be attributed to the fact that contraceptives were generally used after women had achieved the desired family size. In this respect, the concept of family planning needs to be explained more explicitly in the community rather than merely giving knowledge about contraceptives.

Users and non-users both shared the incidences of unplanned pregnancies where they either opted for the abortion or continued with the pregnancy. Some women shared that they didn’t know about the available abortion services in hospitals and were too scared to ask their families about it. It led some of them to attempt abortion using home remedies and other unsafe practices. This could have disastrous implications as unsafe abortion is a leading cause of maternal deaths and can also result in temporary or lifelong disability [38, 39]. Apart from promoting the use of contraceptives to prevent such unintended pregnancies, it is essential to ensure that if required, women can access safe abortion services without hesitation. It is an important component of reproductive health services and rights that should be absolutely free from judgement and discrimination [40].

Some non-user women in our study, though well aware of modern contraceptives, preferred traditional methods like withdrawal and safe-period. They were confident of achieving the desired benefits of family planning like spacing and limiting the number of children by using traditional methods. Globally, similar fertility awareness methods (calendar-based/symptom-based method) have been used to achieve family planning [41–43]. They are less effective in comparison to other modern methods but have cultural acceptance. The quantitative data indicated that nearly 6% were using traditional methods of family planning (not in the table). Women prefer these methods due to various reasons like religious beliefs, health issues and fear of side effects of modern contraceptives as found in our study. A wide range of contraceptives to choose from can be introduced to women worried about side effects and other health-related issues. Alongside, respecting the choice of women, we can also provide them with more information on the menstrual cycle and ovulation period to make them more informed about their bodies.

Fear of side effects of modern contraceptives was quoted extensively in our interviews. Similar findings have been reported by other studies as well [44–46]. The widespread misconception surrounding contraceptives in the community influenced the decision of women who had never tried any modern method. Some of the respondents became non-user after experiencing some complications post-contraceptive uptake. This emphasizes the importance of follow-up with women when they first use contraceptives. Initial reactions of different methods can be explained in detail so women know ‘what to expect’. It is important that they take a well-informed decision about continuing with the method or changing it. We found that door-to-door counselling and frank conversations by the project’s frontline workers helped in dismantling the misconceptions. Women felt confident and were able to decide about using/continuing with modern contraceptives. Our quantitative data also indicated that the uptake of contraceptives was more among women who came in contact with NGOs or the public health system. This finding reverberates with other studies suggesting that support during method selection, information on possible side effects and counselling can significantly increase the use of modern contraceptives [47, 48]. Frontline workers can be equipped with contraception-related Information, Education and Communication (IEC) tools and counselling skills.

Similar to other studies from urban informal settlements, our quantitative data suggested that women’s education was positively associated with contraceptive use [25, 26, 49]. We recognize that in our context, it was also about reproductive and sexual autonomy which enabled women to make decisions for their health regardless of their level of education, a finding similar to other studies [50–52]. In our qualitative sample, one woman with more than 10 years of schooling wanted to use modern contraceptives but was unable to because of family restrictions. Husband’s education was also not significantly associated with the use of contraceptives. Data indicated that a family’s socioeconomic position was positively associated with the use of modern contraceptives like in other studies [25, 26]. A few respondents were involved in home-based informal work but largely women were financially dependent on their husbands. As the primary earner and decision-maker in the family, the husband’s awareness regarding contraceptives was an important factor in its uptake. In our study, we did not collect data from the husbands of our participants but from our interviews, we found that spousal communication influenced the use of contraceptives which is similar to what we found in other studies [53–55]. Husbands with better awareness regarding modern contraceptives either adopted methods themselves or conversed with their wives openly about family planning. Such couples either planned their family just after marriage or after the first few instances of unplanned pregnancies which forced them to decide in favour of the use of contraceptives. Family planning programs should ensure the participation of husbands in awareness and counselling sessions with an aim to improve spousal communication regarding contraceptive use. Other users in a family or role models in the community were also an important factor that helped women in the uptake of contraceptives. Family planning programs can build the capacity of these people for better outreach and negate the widespread misconceptions about modern contraceptives in the community.

In our interaction with both users and non-users, we found that family planning was a taboo subject. The shame associated with it affects information seeking and sharing among young women. With no provision of adolescent sexual and reproductive health education, women enter marriage mostly without any information. Post-marriage societal norms of early childbearing and completing family lead them to postpone the use of contraceptives till they want no more children. During our discussion regarding family size, religion came out as an important phenomenon similar to other studies [56, 57]. But our quantitative data did not show any significant relation. Women justified the use or non-use of modern contraceptives based on their beliefs shaped by their own interpretation of religion [58, 59]. Muslim women preferred reversible methods of contraceptives and had doubts related to terminal methods as it limits pregnancies which was not allowed by their religion.

Family planning has the potential to accelerate the country’s progress towards SDGs. But, any further increase in contraceptive prevalence rate and reduction in unmet need will need contextualized strategies. Table 5 presents the key learning for family planning programs from our study. In summary, our study highlighted that for most of the women in our community, becoming a user or a non-user of contraceptives depended entirely on the chances of her encounter with influencing factors. Women enter marriage quite early and without proper information related to sexual and reproductive health. In this situation, they are not in a position to make decisions and protect their rights on their own. It is well-known that information related to reproductive rights and contraceptive choices is fundamental to empowering individuals to make their reproductive health decisions [40]. It is important to inform women about their reproductive rights and equally important is to empower them to practice these rights. It can be done by increasing women’s age at marriage and continued promotion of women’s formal education which will enable them to take a conversant decisions about their health. We must strengthen the sexual and reproductive health component of adolescent health education. Apart from giving information about reproductive health rights, it will also help in creating an ecosystem where couples can have an open and frank discussion regarding family planning.

**Table 5:** Key recommendations.

Findings	Recommendations
***Perception about modern contraceptives***	
• Women were aware of modern contraceptives but not all thought modern methods were essential for family planning	• Promote women’s education and awareness related to family planning and the benefits of modern contraceptives
• Some non-users practiced traditional methods like withdrawal and safe period, believing them to be a better method free from any side effects	• Help increase awareness about the menstrual cycle and ovulation period to traditional methods users, explain the risk of unintended pregnancies and available choices of modern contraceptives
***Fear of Side Effects***	
• Women’s perception related to the side effects of modern contraceptives was the major deterrent to use, which was either based on personal experience or hearsay	• Negate myths and misconceptions related to modern contraceptives, follow-up and suggestions on available choices of contraceptives, counsel and refer in case of side effects
***Spousal awareness and communication***	
• Spousal awareness or communication helped in the early uptake of contraceptives resulting in better spacing between children and limiting their number	• Involve husbands through planned parenthood and couple counselling sessions
***Family Support***	
• Women got the support of family members in form of getting information and accompanying them to the hospitals for contraceptive uptake	• Identify role models in families or communities for better outreach and eliminate misconceptions related to modern contraceptives
***Counselling and moral support***	
• Mere suggestion of use did not lead to the uptake of contraceptives. Women needed detailed information, proper counselling, support and follow-up in case of any side effects	• Equip frontline workers with better IEC tools related to modern contraceptives, build counselling skills of frontline workers
***Social and cultural context***	
• Taboo associated with family planning affected information seeking and sharing which delayed contraceptive use	• Promote adolescent sexual and reproductive health education and awareness related to contraceptive use and reproductive rights
• Social norm of early childbearing and completing family restricted or delayed use of contraceptive	
***Religious Beliefs***	
• Influenced the choice of contraceptives	• Approach community/religious leaders for better outreach and awareness generation on family planning

The right-baseds approach to family planning must get translated organically into our communities. It can be brought about by roping in role models, community/religious leaders and health professionals and also by involving people who influence women’s decisions, especially husbands and elder women in the family. Their involvement in family planning awareness sessions will ensure that couples will make better informed and timely decisions. Only availability of contraceptives will not protect women’s right to access family planning services. Family planning programs can achieve much more beyond numerical outcomes by targeting factors that influence women’s ability to practice their right to choose.

## Supporting information

S1 File Qualitative tool–English. (DOCX)

S2 File Qualitative tool–Hindi. (DOCX)

S3 File Quantitative tool–English. (DOCX)

S4 File Quantitative tool–Hindi. (DOCX)
