# Supplementary material for: To use or not to use: Exploring factors influencing the uptake of modern contraceptives in urban informal settlements of Mumbai
Source: PLOS Glob Public Health. 2023 Mar 2;3(3):e0000634. doi: 10.1371/journal.pgph.0000634 (PMC10021173; doi:10.1371/journal.pgph.0000634)
Supplement: S1 File — (DOCX) [file pgph.0000634.s001.docx]

**SNEHA** (**S**ociety for **N**utrition, **E**ducation and **H**ealth **A**ction) [www.snehamumbai.org](http://www.snehamumbai.org)


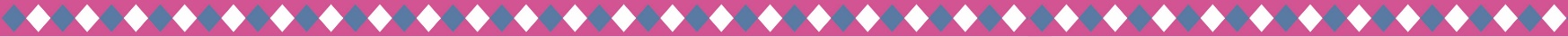


**Family planning and modern contraceptives-related beliefs, awareness and practices**

**Interview guide**

**General Respondent Information**

| Current Age |  |
| --- | --- |
| Age at the time of marriage |  |
| Religion |  |
| Employment |  |
| Spouse employment |  |
| No. of years residing in the community |  |
| Family description |  |
| **Obstetric history** |  |
| Age at the time of first pregnancy |  |
| Gravida (no. of time conceived) |  |
| Parity (no. of time delivered) |  |
| Live birth |  |
| Miscarriage |  |
| Abortion |  |
| Stillbirth |  |

**Family information**

1. Can you please tell us about your family? How many members are there in your family? How many adults and how many children?
2. Is it a joint family or do you live with your husband and children only?
3. How long your family has been in this *basti* (Community)? If it is a migrant family then ask from which state they have come and the reason for leaving their native place. Also, ask for the woman’s migrant status. If she has come to the community after her marriage and from which state?

**Awareness about family planning, modern contraceptives and current practices**

1. Have you ever heard anything about family planning? Can you please tell me what you know about family planning? If the woman says no try asking using popular terms like *parivaar niyojan, nasbandi* etc.
2. What are your views on family planning? What it does? (Probe woman’s views on the need for family planning, what are the benefits or harm of using it?)
3. Can you please tell me if someone wants to do it how can they do it? (Probe her awareness regarding modern contraceptives and if the woman mentions traditional methods like safe period, withdrawal, calendar methods, etc,)
4. Can you please tell me in detail about the methods you mentioned earlier? (Probe types of modern contraceptives and woman’s views related to the use of family planning to get a better understanding of its use for reproductive health or as just a method of stopping /spacing children)
5. How and when you came to know about these methods? (Probe for e.g., Friends, family, health provider, media and which was more influential and why if woman mentions more than one? any personal experience etc.) (was she aware of family planning using contraceptives before her marriage, if yes then who told her about it?)
6. Do you think there is any benefit of using a modern method of contraception? (Probe for benefits that woman perceives will be due to the use of modern contraceptives) What about traditional methods?
7. Are you currently using any method of family planning? (Check if the woman considers traditional methods as a method of family planning)
8. Which method of family planning are you using?

**NON-USER (if woman or her husband uses no method or uses traditional method of family planning)**

1. Can you please tell me about the method you are using?
2. For how long you and your husband have been using the traditional method? What do you think about it? Have you ever tried any modern methods? How was your experience?
3. Do you know about modern contraceptives? Why are you not using any modern contraceptives currently? (Probe for all the reasons individual, family, community) Do you feel any challenges in using modern contraceptives?
4. Do you and your husband ever talk about using modern methods, if not then why?
5. If in the future you want to use it, do you know where to go or whom to contact?

**For Women who used modern contraceptives earlier but are not using them now**

1. Which modern method of contraception were you using earlier? (Probe for which method, for how long, did woman try any other methods)
2. Why did you stop using that method? What happened? (Probe for all the reasons individual, family, community
3. Did you ever tried using any other modern method again? (Try to capture the jump between different methods and reasons for the same)
4. Would you like to use any modern method in the near future, if not, try to understand her views and the reasons for the same.
5. If in the future you want to use it, do you know where to go or whom to contact?

**For women currently using any modern contraceptive**

1. Which modern method of contraception are you using? Is it a temporary or permanent type of method?
2. For how long have you been using this method? Have you been using the same method for this long or have changed between different types of modern contraceptives?
3. If changed, why did you change the method? (Probe for how long each method, and who suggested it)
4. What do you think has supported you in continuing the use of modern contraceptives?
5. Have you ever talked about the use of a modern method of contraception to anybody? if yes whom & why? If not then why?

**Are you currently using any**

******************************************************************
