# Supplementary material for: To use or not to use: Exploring factors influencing the uptake of modern contraceptives in urban informal settlements of Mumbai
Source: PLOS Glob Public Health. 2023 Mar 2;3(3):e0000634. doi: 10.1371/journal.pgph.0000634 (PMC10021173; doi:10.1371/journal.pgph.0000634)
Supplement: S2 File — (DOCX) [file pgph.0000634.s002.docx]

**SNEHA** (**S**ociety for **N**utrition, **E**ducation and **H**ealth **A**ction) I [www.snehamumbai.org](http://www.snehamumbai.org)


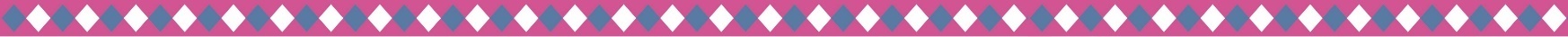


**परिवार नियोजन और आधुनिक गर्भ निरोधकों से संबंधित मान्यताएं, जागरूकता और प्रथाएं**

**साक्षात्कार गाइड**

**सामान्य प्रतिवादी सूचना**

| वर्तमान आयु |  |
| --- | --- |
| शादी के समय उम्र |  |
| धर्म |  |
| रोज़गार |  |
| जीवनसाथी का रोजगार |  |
| समुदाय में रहने वाले वर्षों की संख्या |  |
| परिवार विवरण |  |
| **प्रसूति इतिहास** |  |
| पहली गर्भावस्था के समय उम्र |  |
| ग्रेविडा (कल्पित समय की संख्या) |  |
| समता (वितरित समय की संख्या) |  |
| जीवित पैदाइश |  |
| गर्भपात (miscarriage) |  |
| गर्भपात (abortion) |  |
| स्टीलबर्थ |  |

**परिवार की जानकारी**

1. क्या आप कृपया हमें अपने परिवार के बारे में बता सकते हैं? आपके परिवार में कितने सदस्य हैं? कितने वयस्क और कितने बच्चे?

2. क्या यह संयुक्त परिवार है या आप अपने पति और बच्चों के साथ ही रहती हैं?

3. आपका परिवार इस बस्ती में कब से रहा है? यदि यह एक प्रवासी परिवार है तो पूछें कि वे किस राज्य से आए हैं और अपने मूल स्थान को छोड़ने का कारण पूछें। साथ ही महिला की प्रवासी स्थिति के बारे में पूछें। यदि वह अपनी शादी के बाद समुदाय में आई है और किस राज्य से आई है?

**परिवार नियोजन, आधुनिक गर्भ निरोधकों और वर्तमान प्रथाओं के बारे में जागरूकता**

1. क्या आपने कभी परिवार नियोजन के बारे में कुछ सुना है? क्या आप कृपया मुझे बता सकते हैं कि आप परिवार नियोजन के बारे में क्या जानते हैं? यदि महिला कहती है कि परिवार नियोजन जैसे लोकप्रिय शब्द का उपयोग करके पूछने की कोशिश न करें

2. परिवार नियोजन के बारे में आपके क्या विचार हैं? यह क्या करता है? (परिवार नियोजन की आवश्यकता पर उनके विचारों की जांच करें, लाभ या हानि क्या हैं?)

3. क्या आप कृपया मुझे बता सकते हैं कि अगर कोई इसे करना चाहता है तो वे इसे कैसे कर सकते हैं? (आधुनिक गर्भ निरोधकों के बारे में उसकी जागरूकता की जांच करें और यदि महिला पारंपरिक तरीकों जैसे सुरक्षित अवधि, निकासी, कैलेंडर विधियों आदि के बारे में इसका उल्लेख करती है,)

4. क्या आप मुझे पहले बताए गए तरीकों के बारे में विस्तार से बता सकते हैं? (आधुनिक गर्भ निरोधकों के प्रकार और परिवार नियोजन के उपयोग से संबंधित उनके विचार प्रजनन स्वास्थ्य के लिए इसके उपयोग की बेहतर समझ प्राप्त करने के लिए या बच्चों को रोकने/दूर करने की एक विधि के रूप में)

5. आपको इन तरीकों के बारे में कैसे और कब पता चला? (उदाहरण के लिए जांच। मित्र, परिवार, स्वास्थ्य प्रदाता, मीडिया और जो अधिक प्रभावशाली था और यदि महिला एक से अधिक का उल्लेख क्यों करती है? कोई व्यक्तिगत अनुभव इत्यादि) (अपनी शादी से पहले गर्भ निरोधकों का उपयोग कर परिवार नियोजन के बारे में पता था, यदि हां तो किसने उसके बारे में?)

6. क्या गर्भनिरोधक की आधुनिक पद्धति का उपयोग करने से आपको कोई लाभ है? (उन लाभों की जांच करें जो महिला को लगता है कि एमएमसी के उपयोग के कारण होगा) पारंपरिक तरीकों के बारे में क्या?

7. क्या आप वर्तमान में परिवार नियोजन की किसी विधि का प्रयोग कर रहे हैं? (जांच करें कि क्या महिला पारंपरिक तरीकों को परिवार नियोजन का एक तरीका मानती है)

8. आप परिवार नियोजन की किस विधि का प्रयोग कर रहे हैं?

**गैर-उपयोगकर्ता (यदि महिला या उसका पति परिवार नियोजन की कोई विधि या पारंपरिक पद्धति का उपयोग नहीं करता है)**

1. क्या आप कृपया मुझे उस विधि के बारे में बता सकते हैं जिसका आप उपयोग कर रहे हैं?

2. आप और आपके पति कितने समय से पारंपरिक पद्धति का उपयोग कर रहे हैं? आपने इस बारे में क्या सोचा? क्या आपने कभी कोई आधुनिक तरीका आजमाया है? आपका अनुभव कैसा था?

3. क्या आप आधुनिक गर्भ निरोधकों के बारे में जानते हैं? आप वर्तमान में किसी भी आधुनिक गर्भ निरोधकों का उपयोग क्यों नहीं कर रही हैं? (सभी कारणों से व्यक्तिगत, परिवार, समुदाय के लिए जांच) क्या आप आधुनिक गर्भ निरोधकों का उपयोग करने में कोई चुनौती महसूस करते हैं?

4. क्या आप और आपके पति कभी आधुनिक पद्धति के प्रयोग की बात करते हैं, यदि नहीं तो क्यों?

5. अगर भविष्य में आप इसका इस्तेमाल करना चाहते हैं, तो क्या आप जानते हैं कि कहां जाना है या किससे संपर्क करना है?

**उन महिलाओं के लिए जो पहले आधुनिक गर्भ निरोधकों का उपयोग करती थीं लेकिन अब उनका उपयोग नहीं कर रही हैं**

1. पहले आप गर्भनिरोधक के किस आधुनिक तरीके का इस्तेमाल कर रही थीं? (जांच किस विधि के लिए, कितने समय तक महिला ने अन्य विभिन्न विधियों को भी आजमाया)

2. आपने उस तरीके का इस्तेमाल क्यों बंद कर दिया? क्या हुआ? (व्यक्तिगत, परिवार, समुदाय सभी कारणों से जांच करें

3. क्या आपने फिर कभी किसी अन्य आधुनिक पद्धति का उपयोग करने का प्रयास किया? (उसी के लिए विभिन्न तरीकों और कारणों के बीच की छलांग को पकड़ने की कोशिश करें)

4. क्या आप निकट भविष्य में किसी आधुनिक पद्धति का प्रयोग करना चाहेंगे, यदि नहीं, तो उसके विचारों और उसके कारणों को समझने का प्रयास करें।

5. अगर भविष्य में आप इसका इस्तेमाल करना चाहते हैं, तो क्या आप जानते हैं कि कहां जाना है या किससे संपर्क करना है?

**वर्तमान में किसी भी आधुनिक गर्भनिरोधक का उपयोग करने वाली महिलाओं के लिए**

1. आप गर्भनिरोधक के किस आधुनिक तरीके का उपयोग कर रही हैं? क्या यह एक अस्थायी या स्थायी प्रकार की विधि है?

2. आप कितने समय से इस पद्धति का उपयोग कर रहे हैं? क्या आप लंबे समय से एक ही विधि का उपयोग कर रहे हैं या विभिन्न प्रकार के आधुनिक गर्भ निरोधकों के बीच बदल गए हैं?

3. अगर बदला है, तो आपने तरीका क्यों बदला? (जांच करें कि प्रत्येक विधि कितने समय के लिए है, और किसने इसका सुझाव दिया है)

4. आपको क्या लगता है कि आधुनिक गर्भ निरोधकों के उपयोग को जारी रखने में आपको क्या मदद मिली है?

5. क्या आपने कभी किसी को गर्भनिरोधक के आधुनिक तरीके के इस्तेमाल के बारे में बताया है? , यदि हाँ तो किसको और क्यों ? यदि नहीं तो क्यों?

******************************************************************************************
