# Supplementary material for: To use or not to use: Exploring factors influencing the uptake of modern contraceptives in urban informal settlements of Mumbai
Source: PLOS Glob Public Health. 2023 Mar 2;3(3):e0000634. doi: 10.1371/journal.pgph.0000634 (PMC10021173; doi:10.1371/journal.pgph.0000634)
Supplement: S3 File — (DOCX) [file pgph.0000634.s003.docx]

**SNEHA** (**S**ociety for **N**utrition, **E**ducation and **H**ealth **A**ction) I [www.snehamumbai.org](http://www.snehamumbai.org)


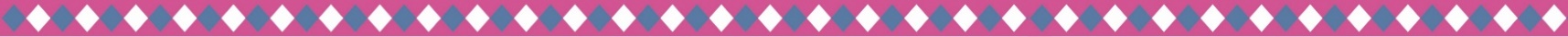


**Endline survey tool, Healthy City Project, SNEHA**

| 1. Write Plot id number (e.g 01) |  |
| --- | --- |
| 1. Enter house hold ID number (e.g 001) |  |
| 1. Is target group present? |  |
|  | Nobody at home |
|  | Nobody lives there |
|  | Target group available |
|  | Target group not available |
| 1. Do you agree to participate in the study? |  |
|  | Yes |
|  | No |
| 1. Target group information. |  |
|  | woman 15-49 years is there |
|  | Mother of 0-2 years old children |
|  | Mother of 2-6 years old children |
| 1. Enter the respondent ID |  |
| **Say to respondent: I am going to ask you about the people who live in your house, starting with you.** | |
| 1. What is the name of the head of household? |  |
| 1. Total household members |  |
| 1. Type of family |  |
|  | Nuclear |
|  | Joint |
| 1. What is your name? |  |
| 1. How old are you? |  |
| 1. How many years of schooling have you had? |  |
| 1. What is your main occupation? |  |
|  | Does not work or looking for work |
|  | Student (school or college) |
|  | Job that does not require skills or training |
|  | Runs machines in a factory, or driver |
|  | Makes products |
|  | Agriculture or fishery worker |
|  | Shop, market, hotel or transport worker |
|  | Junior white collar |
|  | Service sector skilled or technical work |
|  | Professional |
|  | Senior manager or government official |
| 1. Does your husband live here? |  |
|  | Husband lives in household |
|  | Husband died |
|  | Separated, divorced |
|  | Lives elsewhere in India |
|  | Lives abroad |
| 1. How old is he? (If unknown, enter 99) |  |
| 1. How many years of schooling has he had? |  |
| 1. What is his main occupation? |  |
|  | Does not work or looking for work |
|  | Student (school or college) |
|  | Job that does not require skills or training |
|  | Runs machines in a factory, or driver |
|  | Makes products |
|  | Agriculture or fishery worker |
|  | Shop, market, hotel or transport worker |
|  | Junior white collar |
|  | Service sector skilled or technical work |
|  | Professional |
|  | Senior manager or government official |
| 1. What is your religion? |  |
|  | Muslim |
|  | Hindu |
|  | Christian |
|  | Buddhist |
|  | Parsi |
|  | Sikhism |
|  | Jain |
|  | Other |
| 1. How long has the family been living in mumbai ? |  |
| 1. How long have you been living in Mumbai? Enter number of years. |  |
| 1. Where did you live before you moved to Mumbai? |  |
|  | Maharashtra |
|  | Bihar |
|  | Delhi |
|  | Karnataka |
|  | Tamil Nadu |
|  | Uttar Pradesh |
|  | West Bengal |
|  | Other region |
|  | Did you live in a town or a village? |
|  | Town |
|  | Village |
| 1. Did you live there in the city or in the village? |  |
|  | City |
|  | Village |
| 1. Why did you move to Mumbai? |  |
|  | Got married here |
|  | Got a job here |
|  | Better job prospects |
|  | Family moved here |
|  | Better standard of living |
|  | Pregnancy or childbirth |
|  | Other reason |
| 1. How long have you been living in this basti? Enter number of years. |  |
| 1. If you are living less than a year in this settlement, then write the month. |  |
| 1. In the last one year, have you stayed away from this basti for more than one month? |  |
|  | Yes |
|  | No |
| 1. If yes, for how long? |  |
| 1. 4 years ago, we at SNEHA came to visit people in this area and asked them questions list these ones. Do you remember giving an interview like this 4 years ago? |  |
|  | Yes |
|  | No |
| 1. Do your family own or rent your home? |  |
|  | Own |
|  | Rent |
| 1. Do you have a ration card? |  |
|  | Yes |
|  | No |
| 1. What colour is your ration card? |  |
|  | White |
|  | Yellow |
|  | Orange |
|  | Pink |
| 1. Is your name there in the ration card? |  |
|  | Yes |
|  | No |
| 1. select the type of house the respondent lives in |  |
|  | Pucca |
|  | Semi-pucca |
|  | Kaccha |
| 1. Do you own any of the following household items? |  |
|  | Mattress |
|  | Pressure cooker |
|  | Gas cylinder |
|  | Stove |
|  | Chair |
|  | Bed |
|  | Table |
|  | Clock |
|  | Electric fan |
|  | Mixer |
|  | Radio |
|  | Phone (landline or mobile) |
|  | Fridge |
|  | TV |
|  | Bicycle |
|  | Two-wheeler |
|  | Car |
|  | AC |
|  | Computer |
|  | Internet |
|  | Sewing machine |
|  | Washing machine |
| 1. What type of electricity supply does your home have? |  |
|  | None |
|  | Metered, family pay bill |
|  | Pay landlord |
|  | Rent |
|  | Sharing illegal electricity |
|  | Other |
|  | Specify other type of electricity supply |
| 1. What fuel do you use for cooking? |  |
|  | Wood, charcoal, dung |
|  | Kerosene, LPG |
|  | LPG |
|  | Electricity |
|  | Does not cook at home |
| **Wash** | |
| 1. What is the main source of drinking-water for members of your household? |  |
|  | Piped water into dwelling |
|  | Piped water to yard plot |
|  | Public tap standpipe |
|  | Bottled water |
|  | Tanker truck |
|  | Community RO Plant |
|  | Other |
| 1. Do you treat your water in any way to make it safer to drink? |  |
|  | Yes |
|  | No |
|  | Dont know |
| 1. What do you usually do to the water to make it safer to drink? |  |
|  | Boil |
|  | Add bleach/chlorine |
|  | Alum |
|  | Electronic purifier |
|  | Strain it through a cloth |
|  | Use a water filter (ceramic, sand, composite) |
|  | Let it stand and settle |
|  | Other (specify) |
|  | Don’t know |
| 1. What kind of toilet facility do members of your household usually use? |  |
|  | Flush/pour flush |
|  | Private/inside home flush toilet |
|  | No facilities or field or road |
|  | Other (specify) |
| 1. Do you share this facility with other households? |  |
|  | Yes |
|  | No |
| 1. How many households use this toilet facility? (If don't know enter 99) |  |
| **### (Information for a child under the age of two years) ###** | |
| 1. The last time [the baby] passed stools, what was done to dispose of the stools? (Ask for youngest child under 2 years ) |  |
|  | No 2 years or below 2 years child |
|  | Child used toilet latrine |
|  | Put, rinsed into toilet or latrine (sanitary) |
|  | Put/rinsed into drain or ditch |
|  | Thrown into garbage |
|  | Buried (sanitary) |
|  | Left in the open |
|  | Other (specify) |
|  | Dont Know |
| **# Maternal history**    Now I would like to ask you about all the pregnancies you had6 | |
| 1. How old were you when you first got married? |  |
| 1. How many years have you been married? |  |
| **Number of pregnancies** |  |
| 1. How many times did you get pregnant : |  |
|  | live_birth |
|  | Still birth |
|  | Abortion |
|  | Miscarriage |
| 1. Twins baby? |  |
|  | Yes |
|  | No |
| 1. How old were you when you became pregnant for the first time? |  |
| 1. Have any of your children died before the age of 5 years? (If not, then write 0) |  |
| 1. How many times did you get pregnant in the last 5 years? |  |
| 1. Gravida_outcome |  |
| 1. first pregnancy |  |
|  | Live birth |
|  | Still birth |
|  | abortion |
|  | Miscarriage |
| 1. First Delivery date |  |
| 1. second pregnancy |  |
|  | Live birth |
|  | Still birth |
|  | abortion/miscarriage |
|  | Miscarriage |
| 1. Second Delivery date |  |
| 1. Third pregnancy |  |
|  | Live birth |
|  | Still birth |
|  | abortion/miscarriage |
|  | Miscarriage |
| 1. Third Delivery date |  |
| 1. Forth pregnancy |  |
|  | Live birth |
|  | Still birth |
|  | abortion/miscarriage |
|  | Miscarriage |
| 1. Fourth Delivery date |  |
| 1. Fifth pregnancy |  |
|  | Live birth |
|  | Still birth |
|  | abortion/miscarriage |
|  | Miscarriage |
| 1. Fifth Delivery date |  |
| 1. Sixth pregnancy |  |
|  | Live birth |
|  | Still birth |
|  | abortion/miscarriage |
|  | Miscarriage |
| 1. Sixth Delivery date |  |
| 1. Details of Under 5 years children age wise  Age of first child Age of second child   Age of third child Age of fourth child Age of fifth child Age of sixth child |  |
| **# Current pregnancy and London Measure of Unplanned Pregnancy** |  |
| 1. Are you pregnant at present? |  |
|  | Yes |
|  | No |
| 1. Did you know LMP date? |  |
|  | Yes |
|  | No |
| 1. ) Enter the date of LMP |  |
| 1. How many months pregnant are you? |  |
| 1. Enter the date of EDD |  |
| 1. current month |  |
| 1. When you got pregnant, did you want to get pregnant at that time? |  |
|  | Yes |
|  | No |
| 1. Did you want to have a baby later on or did you not want any (more) children? |  |
|  | Later |
|  | No more |
| **Child information** |  |
| 1. Have you been pregnant or had a baby in the last 2 years? |  |
|  | Yes |
|  | No |
| 1. Child ID |  |
| 1. child name |  |
| 1. Did she give birth to twins? |  |
|  | Yes |
|  | No |
| 1. Enter the date of birth (When did you have your youngest (surviving) child?) |  |
| 1. Enter the sex of the child |  |
|  | Male |
|  | Female |
| 1. When you got pregnant, did you want to get pregnant at that time? |  |
|  | Yes |
|  | No |
| 1. Did you want to have a baby later on or did you not want any (more) children? |  |
|  | Later |
|  | No more |
| 1. Did you receive any antenatal care when you were pregnant with (NAME)? |  |
|  | Yes |
|  | No |
| 1. Where did you go for ANC registration? |  |
|  | Municipal Health Post |
|  | Municipal Maternity hospital |
|  | Minicipal Hospital |
|  | Private facility |
|  | Govt.Hospital |
|  | Trust Hospital |
|  | Field camp |
|  | Other |
| 1. When was the pregnancy registered? |  |
| 1. you get a card for recording pregnancy related history?  (Ex. Mother Child Protection Card, Immunization card) |  |
|  | Yes |
|  | No |
| 1. you saw or did not see the cards? |  |
| Seen cards |  |
| Card not seen |  |
| Take ANC card photo |  |
| 1. How many times did you receive antenatal care during last pregnancy? |  |
| 1. As part of antenatal care during last pregnancy, were any of the following done during the first ANC visit? |  |
|  | weight measured during antenatal care |
|  | height measured during antenatal care |
|  | abdominal examination during antenatal care |
|  | blood pressure measured during antenatal care |
|  | urine sample during antenatal care |
|  | blood sample during antenatal care |
|  | tetanus toxoid during antenatal care |
|  | deworming |
|  | ultrasound scan during antenatal care |
|  | Nothing |
| 1. Was the baby born in Mumbai or outside? |  |
|  | Mumbai |
|  | outside mumbai |
| 1. Was the baby born in a health facility or at home? |  |
|  | Facility |
|  | Home |
| 1. At which facility did the delivery happen? |  |
|  | Municipal health post |
|  | Municipal Maternity hospital |
|  | Minicipal Hospital |
|  | Private facility |
|  | Govt.Hospital |
|  | Trust Hospital |
|  | Other |
| 1. Which type of delivery you had? |  |
|  | Normal/vaginal |
|  | LSCS |
| 1. Enter the birth weight of the (e.g 2500, If not, then write 9999) |  |
| 1. After your discharged, have you gone to see a doctor? |  |
|  | No |
|  | Within 2 days of childbirth |
|  | Between 3 – 7 days after childbirth |
|  | Between 8 – 42 days after childbirth |
|  | Stayed in hospital for long time after childbirth |
| 1. What was the reason for which you went to this hospital? Select all that apply. |  |
|  | Vaccination |
|  | Maternal Complication |
|  | Contraception advise |
|  | Mother General examination |
|  | Child General examination |
|  | Child complication |
|  | Other |
| # Family planning   Now I am going to ask you about family planning | |
| 1. When did your last menstrual period start? |  |
|  | Less than 6 months ago |
|  | More than 6 months ago |
|  | Before last birth |
|  | Menopause or terminal family planning |
|  | Never menstruated |
| 1. Are you currently doing something or using any method to delay or avoid getting pregnant? |  |
|  | Yes |
|  | No |
| 1. What is the main method you are using? |  |
|  | Female sterilization |
|  | Male sterilization |
|  | Pill |
|  | IUD/loop |
|  | Injectables |
|  | Implants |
|  | Condom/nirodh |
|  | Female condom |
|  | Diaphragm |
|  | Foam/jelly |
|  | Rhythm method |
|  | Withdrawal |
|  | LAM |
| 1. Where did you get this method? |  |
|  | Municipal health post or dispensary |
|  | Municipal maternity home |
|  | Municipal hospital |
|  | Private GP |
|  | Private hospital |
|  | Government hospital |
|  | Medicine shop |
|  | FPAI |
|  | NGO |
|  | CHV etc |
|  | Other |
| 1. Have you ever used emergency contraception pill (pill taken within three days of unprotected sexual intercourse) |  |
|  | Yes |
|  | No |
| 1. How many times have you used emergency contraception? |  |
| 1. Have you ever used anything or tried in any way to delay or avoid getting pregnant in the past? |  |
|  | Yes |
|  | No |
| 1. Has your menstrual period returned since the birth of your last baby? |  |
|  | Yes |
|  | No |
| 1. Now I have some questions about the future. Would you like to have a/another child, or would you prefer not to have any more children? |  |
|  | Have a another child |
|  | Undecided, don’t know |
|  | Says she can’t get pregnant |
|  | No more/none |
| 1. How long would you like to wait from now before the birth of a/another child? |  |
|  | Wait less than 2 years |
|  | Wait more than 2 years |
|  | Other |
|  | Has not decided |
| 1. You have said that you do not want any (more) children. Can you tell me why you are not using a method to prevent pregnancy? Any other reason? |  |
|  | Widow/divorced |
|  | Fertility-related reason |
|  | Not having sex |
|  | Infrequent sex |
|  | Menopause/hysterectomy |
|  | Can’t get pregnant |
|  | Not menstruated since last birth |
|  | Breastfeeding |
|  | Up to God/fatalistic |
|  | Opposition to use: |
|  | Respondent opposed |
|  | Husband opposed |
|  | Others opposed |
|  | Religious prohibition |
|  | Lack of knowledge: |
|  | Knows no method |
|  | Knows no source |
|  | Method-related reasons: |
|  | Side-effects/health concerns |
|  | Lack of access |
|  | Costs too much |
|  | Preferred method not available |
|  | No method available |
|  | Inconvenient to use |
|  | Interferes with body’s normal processes |
|  | Other |
|  | Don’t know |
| **# IYCF** | |
| 1. Was the baby ever breastfed? |  |
|  | Yes |
|  | No |
| 1. How long after birth was put to the breast? Enter number in hours. If &lt;1hr, enter 0. If >24hrs, enter 88. If unknown, enter 99 |  |
| 1. Enter the number of days |  |
| 1. In the first three days after delivery, was given anything to drink other than breast milk? |  |
|  | Yes |
|  | No |
| 1. Which of the following was given to baby in first 3 days? |  |
|  | Only breast milk |
|  | Plain water |
|  | Sugar/glucose water |
|  | Gripe water |
|  | Sugar-salt-water solution |
|  | Other non-milk liquids/juice |
|  | Infant Formula |
|  | Tea |
|  | Honey |
|  | Janam Ghutti |
|  | Other |
| 111. Is still being breastfed? |  |
|  | Yes |
|  | No |
| 112. For how many months was) breastfed? |  |
| 113. How many times did you breastfeed last night between sunset and sunrise? |  |
| 114. How many times was ( <output value="#form/childname" /> ) breastfed yesterday during the daylight hours? |  |
| 115. Did child received anything from a bottle with a nipple yesterday or last night? |  |
|  | Yes |
|  | No |
| 116. Next I would like to ask you about some liquids that may have had yesterday during the day or at night. Did have any of the following? |  |
|  | Plain water |
|  | Yes |
|  | No |
|  | Dont Know |
|  | Infant formula such as Lactogen |
|  | Yes |
|  | No |
|  | Dont Know |
|  | Other milk such as tinned, powdered or fresh animal milk |
|  | Yes |
|  | No |
|  | Dont Know |
|  | Lassi, chaas or other yoghurt drinks |
|  | Yes |
|  | No |
|  | Dont Know |
| 117. How many times did (NAME) have milk of any kind yesterday during the day or at night? If 7 or more times, record 7 |  |
|  | Fruit juice |
|  | Yes |
|  | No |
|  | Dont Know |
|  | Clear broth |
|  | Yes |
|  | No |
|  | Dont Know |
|  | Tea or coffee |
|  | Yes |
|  | No |
|  | Dont Know |
|  | Cold drinks |
|  | Yes |
|  | No |
|  | Dont Know |
| 118. Any other liquids |  |
|  | Yes |
|  | No |
|  | Dont Know |
| 119. Have ever been given any kind of solid foods, or semi-solid food? |  |
|  | Yes |
|  | No |
|  | Dont Know |
| 120. I would like to ask you about the food (NAME) ate yesterday during the day or at night, either separately or combined with other foods. Did eat any of the following? |  |
|  | Commercial baby food |
|  | Yes |
|  | No |
|  | Dont Know |
| 121. Porridge, bread, roti, chapatti, rice, idli, or any other foods made from grains |  |
|  | Yes |
|  | No |
|  | Dont Know |
| 122. Pumpkin, carrots, sweet potatoes that are yellow or orange inside |  |
|  | Yes |
|  | No |
|  | Dont Know |
| 123. White potatoes, white yams, cassava, or any other foods made from roots |  |
|  | Yes |
|  | No |
|  | Dont Know |
| 124. Dark green leafy vegetables |  |
|  | Yes |
|  | No |
|  | Dont Know |
| 125. Ripe mangoes, papayas, cantaloupe or jackfruit |  |
|  | Yes |
|  | No |
|  | Dont Know |
| 126. Other fruits or vegetables |  |
|  | Yes |
|  | No |
|  | Dont Know |
| 127. Liver, kidney, heart or other organ meats |  |
|  | Yes |
|  | No |
|  | Dont Know |
| 128. Chicken, duck or other birds |  |
|  | Yes |
|  | No |
|  | Dont Know |
| 129. Other meat |  |
|  | Yes |
|  | No |
|  | Dont Know |
| 130. Eggs |  |
|  | Yes |
|  | No |
|  | Dont Know |
| 131. Fresh or dried fish or shellfish |  |
|  | Yes |
|  | No |
|  | Dont Know |
| 132. Foods made from beans, peas or lentils? |  |
|  | Yes |
|  | No |
|  | Dont Know |
| 133. Nuts |  |
|  | Yes |
|  | No |
|  | Dont Know |
| 134. Cheese, yoghurt or other milk products |  |
|  | Yes |
|  | No |
|  | Dont Know |
| 135. Food made with oil, fat, ghee or butter |  |
|  | Yes |
|  | No |
|  | Dont Know |
| 136. Sugary foods such as chocolates, sweets, candies, pastries, cakes or biscuits,ice-cream |  |
|  | Yes |
|  | No |
|  | Dont Know |
| 137. Nalli or wafers |  |
|  | Yes |
|  | No |
|  | Dont Know |
| 138. Vada Pav,Samosa, Bhajjiya |  |
|  | Yes |
|  | No |
|  | Dont Know |
| 139. noodles |  |
|  | Yes |
|  | No |
|  | Dont Know |
| 140. Chinese bhel/pakoda |  |
|  | Yes |
|  | No |
|  | Dont Know |
| 141. Bread/PavToast/butter/khaari/paanipuri |  |
|  | Yes |
|  | No |
|  | Dont Know |
| 142. Any other solid or semi-solid food |  |
|  | Yes |
|  | No |
|  | Dont Know |
| 143. How many times did eat solid, semi-solid, or soft foods other than liquids yesterday during the day or at night? |  |
|  |  |
| **Immunization** |  |
| 144. Does woman have immunization card? |  |
|  | Yes |
|  | No |
| 145. Does woman have immunization card? |  |
|  | Seen cards |
|  | Card not seen |
| Take a card image |  |
| 146. BCG (after birth) |  |
|  | Yes |
|  | No |
|  | Don't Know |
| 147. Polio 0 (after birth) |  |
|  | Yes |
|  | No |
|  | Don't Know |
| 148. Hepatitis B (after birth) |  |
|  | Yes |
|  | No |
|  | Don't Know |
| 149. Polio -1 (after 1.5 month) |  |
|  | Yes |
|  | No |
|  | Don't Know |
| 150. DPT-1 (after 1.5 month) |  |
|  | Yes |
|  | No |
|  | Don't Know |
| 151. Hepatitis(B-1) (after 1.5 month) |  |
|  | Yes |
|  | No |
|  | Don't Know |
| 152. IPV- 1 (after 1.5 month) |  |
|  | Yes |
|  | No |
|  | Don't Know |
| 153. Penta - 1 (after 1.5 month) |  |
|  | Yes |
|  | No |
|  | Don't Know |
| 154. Polio-2 (2.5 months) |  |
|  | Yes |
|  | No |
|  | Don't Know |
| 155. DPT-2 (2.5 months) |  |
|  | Yes |
|  | No |
|  | Don't Know |
| 156. Hepatitis(B-2) (2.5 months) |  |
|  | Yes |
|  | No |
|  | Don't Know |
| 157. Penta - 2 (2.5 months) |  |
|  | Yes |
|  | No |
|  | Don't Know |
| 158. Polio-3 (3.5 months) |  |
|  | Yes |
|  | No |
|  | Don't Know |
| 159. DPT 3 (3.5 months) |  |
|  | Yes |
|  | No |
|  | Don't Know |
| 160. Hepatitis(B-3) (3.5 months) |  |
|  | Yes |
|  | No |
|  | Don't Know |
| 161. IPV- 2 (3.5 months) |  |
|  | Yes |
|  | No |
|  | Don't Know |
| 162. Penta - 3 (3.5 months) |  |
|  | Yes |
|  | No |
|  | Don't Know |
| 163. Measles (After 9 month) |  |
|  | Yes |
|  | No |
|  | Don't Know |
| 164. MMR (after 15 month) |  |
|  | Yes |
|  | No |
|  | Don't Know |
| 165. Where did most of the immunization be applied? |  |
|  | Municipal health post |
|  | Municipal hospital |
|  | Private practitioner |
|  | Private hospital |
|  | Government hospital |
|  | Urban health centre |
|  | Outreach camp |
| **Infant illness and treatment** | |
| 166. Has had diarrhea in the last 15 days? |  |
|  | Yes |
|  | No |
| 167. Did you seek advice or treatment for the diarrhea from any source? |  |
|  | Yes |
|  | No |
| 168. Has “childname" been ill with a fever in the last 15 days? |  |
|  | Yes |
|  | No |
| 169. Has “childname" been ill with a cough in the last 15 days? |  |
|  | Yes |
|  | No |
| 170. When "childname" had the illness with a cough, did s/he breathe faster than usual with short, rapid breaths or have difficulty breathing? |  |
|  | Yes |
|  | No |
| 171. When "childname" had the illness, did she/he have a problem in the chest or a blocked or runny nose? |  |
|  | Chest only |
|  | Nose only |
|  | Both |
|  | Don't Know |
| 172. Did you seek advice or treatment for the illness from any source? |  |
|  | Yes |
|  | No |
| 173. Has “childname" had any other illness in the last month? |  |
|  | Yes |
|  | No |
| 174. What sort of illness? |  |
|  | Rash |
|  | Vomiting |
|  | Skin infection |
|  | Ear infection |
|  | Jaundice |
|  | Stomach problem |
|  | Urine problem |
|  | Fits or seizures |
|  | Injury |
|  | other |
| **Intervention processes** |  |
| 175. Are you aware about SNEHA in your plot? |  |
|  | Yes |
|  | No |
| 176. Do you know on which subjects SNEHA works? |  |
|  | Yes |
|  | No |
| 177. What sort of information is given? |  |
|  | Family planning |
|  | Immunization |
|  | Growth monitoring |
|  | Nutrition and health education |
|  | Counseling for violence |
|  | Access care |
|  | Referral services |
|  | other |
| 178. In the last one year have you or your family got any service from the SNEHA Centre? |  |
|  | Yes |
|  | No |
| 179. What were the services received? |  |
|  | Immunization |
|  | Family planning |
|  | Growth monitoring |
|  | Nutrition and health education |
|  | Counseling for violence |
|  | Acces to health facility |
|  | Referral services |
|  | other |
| 180. Did you or your family ever visited SNEHA Centre in last one year? |  |
|  | Yes |
|  | No |
| 181. Why did you or your family visited there? |  |
|  | Growth monitoring |
|  | Group meetings |
|  | Events |
|  | Anemia Camp |
|  | Violence case registration. |
|  | Immunization camp |
|  | Other |
|  | CAG Meeting |
| 182. Has anyone from the SNEHA Centre visited you or your family in last one year? |  |
|  | Yes |
|  | No |
| 183. How often does she visit you? |  |
|  | Weekly |
|  | Fortnightly |
|  | Monthly |
|  | Bi-monthly |
|  | Rarely |
|  | Never |
| **(In last one year) Information about Sneha Center** | |
| 184. In the last one year have you or your family members ever participated in any activity of the SNEHA Centre? |  |
|  | Yes |
|  | No |
| 185. In which activities did you or your family members participated? |  |
|  | Group meetings |
|  | Parents Meeting |
|  | Male Meetings |
|  | Events |
|  | Anemia camp |
|  | Visits to NGOs, Health Posts |
|  | Other |
| 186. Which event did you participate? |  |
|  | Godbharai |
|  | Ushtavan |
|  | Couple Activity |
|  | Monthly Violence campaign |
|  | Cooking demonstration |
|  | Other |
|  | Breastfeeding week |
|  | Nutrition week |
|  | CAG capacity building |
|  | 16 days activism |
| 187. Was this information useful for you? |  |
|  | Yes |
|  | No |
|  | Dont know |
| 188. Do you know of anyone in the area who volunteers with SNEHA? |  |
|  | Yes |
|  | No |
|  | I am a volunteer |
|  | Don’t know |
| 189. Did a SNEHA volunteer ever visited you in last one year? |  |
|  | Yes |
|  | No |
| **ICDS and BMC services** | |
| 189. During last one year, have you or your children received any benefits from Anganwadi/ICDS centre |  |
|  | Yes |
|  | No |
|  | Supplementary nutrition |
|  | Children 6 months to 6 years |
|  | Pregnant women |
|  | Lactating mothers |
|  | Nothing |
|  | Growth Monitoring |
|  | Children 6 months to 6 years |
|  | Pregnant women |
|  |  |
|  |  |
|  |  |
|  |  |
|  |  |
|  |  |
|  |  |
|  |  |
|  |  |
|  |  |
|  |  |
|  |  |
|  |  |
|  |  |
|  |  |
| 190. Referral by ICDS |  |
|  | Yes |
|  | No |
| 191. In last one year, have you or your children received any benefits from BMC |  |
|  | Yes |
|  | No |
| 192. In last one year, did you receive any services from BMC? If yes, what were the services received? |  |
|  | Antenatal care |
|  | Delivery care |
|  | Postnatal care |
|  | Family planning |
|  | Mother Morbidity Care |
|  | Health worker visited |
|  | Other |
|  | Nothing |
|  | Malnutrition |
|  | Immunization |
|  | Deworming |
|  | Health camps |
|  | Child Morbidity Care |
| 193. In the last 1 year have you or your family got any service from an NGO other than SNEHA? |  |
|  | Yes |
|  | No |
| 194. Which NGO |  |
|  | FHF |
|  | FPAI |
|  | PATH |
|  | Arman |
|  | NASEOH |
|  | Apnalaya |
|  | Ameri Cares |
|  | Vision Rescue |
|  | Religious Trust |
|  | Satya Sai Trust |
|  | Doctor’s for you |
|  | Reliance Foundation |
|  | Niramaya Foundation |
|  | Stree Mukti Sangathana |
|  | Other |
| 195. Name of other NGO |  |
| 196. Was the program officer present during this interview? |  |
|  | No |
|  | aaaa |
|  | bbbb |
| Say to respondent: We have finished the questionnaire. Thank you for your time. Is there anything you would like to ask me? |  |
