# Supplementary material for: To use or not to use: Exploring factors influencing the uptake of modern contraceptives in urban informal settlements of Mumbai
Source: PLOS Glob Public Health. 2023 Mar 2;3(3):e0000634. doi: 10.1371/journal.pgph.0000634 (PMC10021173; doi:10.1371/journal.pgph.0000634)
Supplement: S4 File — (DOCX) [file pgph.0000634.s004.docx]

**SNEHA** (**S**ociety for **N**utrition, **E**ducation and **H**ealth **A**ction) I [www.snehamumbai.org](http://www.snehamumbai.org)


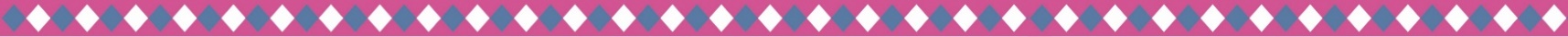


**Endline survey tool, Healthy City Project, SNEHA**

**एंडलाइन सर्वे टूल, स्वस्थ शहर परियोजना स्नेहा**

| 1. प्लॉट आईडी नंबर लिखें (जैसे 01) |  |
| --- | --- |
| 1. घर क्रमांक लिखिए (जैसे 001) |  |
| 1. क्या लक्ष्य समूह मौजूद है? |  |
|  | घर पर कोई नहीं |
|  | वहां कोई नहीं रहता |
|  | लक्षित महिला उपलब्ध है\| |
|  | लक्ष्य महिला मौजूद नहीं\| |
| 1. क्या आप इस सर्वे में हिस्सा लेने के लिए राज़ी है? |  |
|  | हाँ |
|  | ना |
| 1. लक्ष्य महिला की जानकारी |  |
|  | 15-49 उम्र की महिला यहाँ रहती है |
|  | 0- 2 साल के उम्र के बच्चे यहाँ रहते है |
|  | 2-6 साल के बच्चों की माँ |
| 1. जानकारी देनेवाले का आइ डी नंबर लिखिए |  |
| **जानकारी देनेवाले को बोलिये : मैं अभी आप से इस घर में रहनेवाले लोगो के बारे में जानकारी लूंगी\| सबसे पहले मैं आप से शुरू करुँगी\|** | |
| 1. इस घर के मुखिया का नाम क्या है? |  |
| 1. परिवार की कुल संख्या |  |
| 1. परिवार के प्रकार |  |
|  | विभक्त (separate) |
|  | संयुक्त (Joint Family) |
| 1. आप का नाम क्या है? |  |
| 1. आप की उम्र कितनी है ? (उम्र 15 से 49 के अंदर होनी चाहिए ) |  |
| 1. आप ने कहाँ तक पढाई की है ? |  |
| 1. आप क्या काम करते हैं ? |  |
|  | काम नहीं करते , काम ढूढ़ रहे है |
|  | विद्यार्थी |
|  | काम जिसे करने के लिए विशेष ट्रेनिंग और डिग्री कि जरुरत नहीं है |
|  | फैक्ट्री में काम करनेवाला , या ड्राईवर |
|  | सामान बनाते हैं |
|  | किसान या मछली पकड़नेवाला |
|  | दूकान, मार्किट, होटल या ट्रांसपोर्ट में काम करते हैं |
|  | ऑफिस वर्क : कंप्यूटर, टेलिकॉम, कॉल सेंटर |
|  | कुशल या तकनिकी काम , (सामान नहीं बनाते है) |
|  | काम जिसे करने के लिए विशेष ट्रेनिंग और डिग्री कि जरुरत है |
|  | ऊँचे पद का अधिकारी या सरकारी अधिकारी |
| 1. क्या आपके पति आपके साथ रहते है? |  |
|  | पति घर में साथ रहते है |
|  | पति की मृत्यु हो गयी है |
|  | अलग रहते है / तलाकशुदा |
|  | भारत में कहीं और रहते है |
|  | विदेश में रहते है |
| 1. उनकी उम्र कितनी है ? (अगर पता नहीं है तो 99 लिखिए) |  |
| 1. उन्होंने कहाँ तक पढ़ाई की है ? |  |
| 1. वह क्या काम करते है? |  |
|  | काम नहीं करते , काम ढूढ़ रहे है |
|  | विद्यार्थी |
|  | काम जिसे करने के लिए विशेष ट्रेनिंग और डिग्री कि जरुरत नहीं है |
|  | फैक्ट्री में काम करनेवाला , या ड्राईवर |
|  | सामान बनाते हैं |
|  | किसान या मछली पकड़नेवाला |
|  | दूकान, मार्किट, होटल या ट्रांसपोर्ट में काम करते हैं |
|  | ऑफिस वर्क : कंप्यूटर, टेलिकॉम, कॉल सेंटर |
|  | कुशल या तकनिकी काम , (सामान नहीं बनाते है) |
|  | काम जिसे करने के लिए विशेष ट्रेनिंग और डिग्री कि जरुरत है |
|  | ऊँचे पद का अधिकारी या सरकारी अधिकारी |
| 1. आप का धर्म क्या है ? |  |
|  | मुस्लिम |
|  | हिन्दू |
|  | क्रिश्चियन |
|  | बौद्ध |
|  | पारसी |
|  | सिखधर्म |
|  | जैन |
|  | अलग |
| 1. आपका परिवार मुंबई में कब से रहता है ? (नंबर साल में लिखिए \| अगर 1 साल से कम है तो 0 लिखिए \| अगर जनम से रहते है तो 99 लिखिए ) |  |
| 1. आप मुंबई में कितने दिनों से रहते है? (नंबर साल में लिखिए\| अगर 1 साल से कम है तो 0 लिखिए\| अगर जनम से रहते है तो 99 लिखिए ) |  |
| 1. मुंबई में आने से पहले आप कहाँ रहते थे? |  |
|  | महाराष्ट्र |
|  | बिहार |
|  | दिल्ली |
|  | कर्नाटक |
|  | तमिलनाडु |
|  | उत्तर प्रदेश |
|  | वेस्ट बंगाल |
|  | अलग राज्य |
| 1. आप वहाँ शहर में रहते थे या गाव में? |  |
|  | शहर |
|  | गाँव |
| 1. आप मुंबई में रहने के लिए क्यों आए ? |  |
|  | यहाँ पर शादी हुई |
|  | यहाँ पर नौकरी मिली |
|  | बेहतर नौकरी मिलने की संभावना |
|  | परिवार यहाँ रहने आया |
|  | रहन सहन का बेहतर स्तर |
|  | प्रेगनेंसी या डिलीवरी के लिए आयी |
|  | अलग |
| 1. आप इस बस्ती में कितने सालों से रहते है? वर्षों की संख्या दर्ज करें। |  |
| 1. आप इस बस्ती में अगर एक साल से कम रह रहे हो तो महिना लिखिए. |  |
| 1. पिछले एक साल में, क्या आप इस बस्ती से बाहर एक से ज्यादा महिनों के लिए रहने के लिए गये थे ? |  |
|  | हाँ |
|  | ना |
| 1. यदि हाँ, तो कितने महिनों के लिए ? |  |
| 1. 4 साल पहले, हम SNEHA में इस क्षेत्र के लोगों से मिलने आए थे और उनसे सवाल पूछे थे। क्या आपको 4 साल पहले इस तरह का इंटरव्यू देना याद है? |  |
|  | हां |
|  | ना |
| 1. आप का घर खुद का या भाड़े का है ? |  |
|  | खुद का |
|  | भाड़े का |
| 1. क्या आप का राशन कार्ड है? |  |
|  | हाँ |
|  | ना |
| 1. राशन कार्ड कौन से रंग का है? |  |
|  | सफ़ेद |
|  | पीला |
|  | नारंगी |
|  | गुलाबी |
| 1. क्या आपका नाम राशन कार्ड में है? |  |
|  | हाँ |
|  | ना |
| 1. घर को देख कर लिखिए\| |  |
|  | पक्का |
|  | आधा पक्का |
|  | कच्चा |
| 1. क्या आपके पास यह सब घर की चीजे है |  |
|  | गद्दा |
|  | प्रेशर कुकर |
|  | गॅस सिलिंडर |
|  | स्टोव |
|  | कुर्सी |
|  | बेड |
|  | टेबल |
|  | घड़ी |
|  | इलेक्ट्रिक फैन |
|  | मिक्सर |
|  | रेडियो |
|  | फ़ोन (लैंडलाइन या मोबाइल) |
|  | फ्रिज |
|  | टीवी |
|  | साइकिल |
|  | टु-व्हीलर |
|  | कार |
|  | ए.सी. |
|  | कंप्यूटर |
|  | इन्टरनेट |
|  | सिलाई मशीन |
|  | वॉशिंग मशीन |
| 1. आप के घर के बिजली का सप्लाई कैसा है? |  |
|  | नहीं है |
|  | मीटर का है, परिवारवाले बिल भरते है |
|  | मकान मालिक को देते है |
|  | भाड़े का |
|  | गैरकानूनी बिजली \| |
|  | अलग |
|  | अगर बिजली का सप्लाई ‘अलग’ है तो कैसा है लिखिए\| |
| 1. आप खाना बनाने के लिए कौन सा ईंधन इस्तेमाल करते है ? |  |
|  | लकड़ी, कोयला, गोबर |
|  | केरोसिन |
|  | एल.पी.जी. |
|  | इलेक्ट्रिसिटी |
|  | घर पर खाना नहीं बनाते है |
| **WASH जल और स्वच्छता** | |
| 1. आपके घर के सदस्यों के लिए पीने के पानी का मुख्य स्रोत क्या है? |  |
|  | घर में नल |
|  | घर के बाहर नल |
|  | बस्ती का सामायिक नल |
|  | बोतल का पानी |
|  | टैंकर - ट्रक |
|  | कम्युनिटी RO यंत्र |
|  | अन्य |
| 1. क्या आप पीने का पानी सुरक्षित बनाने के लिए कुछ करते है ? |  |
|  | हाँ |
|  | ना |
|  | पता नहीं |
| 1. पानी सुरक्षित बनाने के लिए आप क्या करते है ? |  |
|  | उबालते है |
|  | ब्लीच / क्लोरीन डालते है |
|  | फिटकिरी |
|  | Electronic purifier |
|  | कपडे से छानते है |
|  | वॉटर फिल्टर (बिना बिजली) |
|  | पानी स्थिर होने देते है |
|  | अन्य (स्पष्ट कीजिये) |
|  | पता नहीं |
| 1. आप लोग किस प्रकार का शौचालय इस्तमाल करते है? |  |
|  | फ्लश |
|  | घर के अंदर फ्लश शौचालय |
|  | कोई सुविधा नहीं/ मैदान /सड़क |
|  | अन्य (स्पष्ट कीजिये) |
| 1. क्या आप इस शौचालय को अन्य घरों के साथ बाटते है? |  |
|  | हाँ |
|  | ना |
| 1. इस शौचालय का इस्तमाल कितने घर करते है ? (अगर पता नहीं है तो 99 लिखिए ) |  |
| **### (दो साल या उससे कम उम्र के बच्चे के बारे मैं जानकारी) ###** | |
| 1. [बच्चे] ने दस्त या संडास किया था तो आपने उसे किस तरह फेका था ?(सबसे छोटे बच्चे के बारे मैं पूछे ? |  |
|  | 2 साल या उससे छोटा बच्चा नहीं है |
|  | बच्चे ने शौचालय का इस्तेमाल किया |
|  | शौचालय में फेका / धोया था |
|  | नाली /गड्डा में फेकते है |
|  | कचरे में फेका था |
|  | जमीन में गाढ़ा था |
|  | खुले में छोड़ा था |
|  | अन्य |
|  | पता नहीं |
| **#( Maternal history) मातृ इतिहास  अभी मैं आप से आपकी प्रेगनेंसी के बारेमें जानकारी लूगी** | |
| 1. जब अपने पहली बार शादी की तो आपकी उम्र क्या थी? |  |
| 1. आपके शादी को कितने साल हुए ? |  |
| **## गर्भधारणा की संख्या ##** |  |
| 1. आप कितनी बार गर्भवती हुई |  |
|  | जीवित जन्म |
|  | मृत बच्चे का जन्म |
|  | गर्भपात किया / करवाया |
|  | गर्भपात हुआ |
| 1. जुड़वां बच्चे है ? |  |
|  | हाँ |
|  | ना |
| 1. पहली गर्भधारणा के समय आपकी उम्र कितनी थी ? |  |
| 1. क्या आप के किसी बच्चे की मौत 5 साल से कम उम्र में हुई है ? (अगर नहीं हुई है, तो 0 लिखिए) |  |
| 1. पिछले 5 साल में आप कितनी बार गर्भवती हुई ?  (अगर गर्भवती नहीं हुई है, तो 0 लिखिए, अगर वह अभी प्रेगनेंट है तो, इस प्रेगनेंसी को शामिल ना करे। |  |
| 1. गर्भधारणा का परिणाम |  |
| 1. पहली गर्भावस्था |  |
|  | जीवित जन्म |
|  | मृत बच्चे का जन्म |
|  | गर्भपात किया / करवाया |
|  | गर्भपात हुआ |
| 1. पहली डिलीवरी की तारीख (अगर गर्भपात किया / करवाया है तो, उसकी गर्भपात की तारीख क्या है ) |  |
| 1. दूसरी गर्भावस्था |  |
|  | जीवित जन्म |
|  | मृत बच्चे का जन्म |
|  | गर्भपात किया / करवाया |
|  | गर्भपात हुआ |
| 1. दूसरी डिलीवरी की तारीख (अगर गर्भपात किया / करवाया है तो, उसकी गर्भपात की तारीख क्या है ) |  |
| 1. तीसरी गर्भावस्था |  |
|  | जीवित जन्म |
|  | मृत बच्चे का जन्म |
|  | गर्भपात किया / करवाया |
|  | गर्भपात हुआ |
| 1. तीसरी डिलीवरी की तारीख (अगर गर्भपात किया / करवाया है तो, उसकी गर्भपात की तारीख क्या है ) |  |
| 1. चौथी गर्भावस्था |  |
|  | जीवित जन्म |
|  | मृत बच्चे का जन्म |
|  | गर्भपात किया / करवाया |
|  | गर्भपात हुआ |
| 1. चौथी डिलीवरी की तारीख (अगर गर्भपात किया / करवाया है तो, उसकी गर्भपात की तारीख क्या है ) |  |
| 1. पांचवी गर्भावस्था |  |
|  | जीवित जन्म |
|  | मृत बच्चे का जन्म |
|  | गर्भपात किया / करवाया |
|  | गर्भपात हुआ |
| 1. पांचवी डिलीवरी की तारीख (अगर गर्भपात किया / करवाया है तो, उसकी गर्भपात की तारीख क्या है ) |  |
| 1. छठवी गर्भावस्था |  |
|  | जीवित जन्म |
|  | मृत बच्चे का जन्म |
|  | गर्भपात किया / करवाया |
|  | गर्भपात हुआ |
| 1. छठवी डिलीवरी की तारीख (अगर गर्भपात किया / करवाया है तो, उसकी गर्भपात की तारीख क्या है ) |  |
| 1. 5 वर्ष से कम उम्र के बच्चों के विवरण पहले बच्चे की उम्र  दूसरे बच्चे की उम्र  तीसरे बच्चे की उम्र  चौथे बच्चे की उम्र  पाचवे बच्चे की उम्र  छठवे बच्चे की उम्र |  |
| **# वर्तमान गर्भावस्था /अनियोजित गर्भावस्था** |  |
| 1. क्या आप अभी पेट से है |  |
|  | हाँ |
|  | ना |
| 1. क्या आपको पिछले माहवारी की तारीख पता है? |  |
|  | हाँ |
|  | ना |
| 1. एल.एम.पी की तारीख लिखिए |  |
|  |  |
| 1. आप का कौन सा महीना चल रहा है? |  |
| 1. इ.डी.डी की तारीख लिखिए |  |
| 1. चालू महिना |  |
| 1. जब आप प्रेगनेंट हुई, उस समय क्या आप प्रेगनेंट रहना चाहती थी? |  |
|  | हाँ |
|  | ना |
| 1. क्या आप को बाद में बच्चा चाहिए था या आप को और बच्चा नहीं चाहिए था? |  |
|  | बाद में |
|  | नहीं चाहिए था |
| **###बचे की जानकारी###** |  |
| 1. क्या पिछले 2 सालों में आप को बच्चा हुआ है? |  |
|  | हाँ |
|  | ना |
| 1. बच्चा आय.डी |  |
| 1. बच्चे का नाम क्या है? |  |
| 1. क्या आप को जुड़वाँ बच्चा हुआ? |  |
|  | हाँ |
|  | ना |
| 1. जन्म की तारीख लिखें |  |
| 1. बच्चे का लिंग |  |
|  | लड़का |
|  | लड़की |
| 1. जब आप प्रेगनेंट हुई, उस समय क्या आप प्रेगनेंट रहना चाहती थी? |  |
|  | हाँ |
|  | ना |
| 1. क्या आप को बाद में बच्चा चाहिए था या आप को और बच्चा नहीं चाहिए था? |  |
|  | बाद में |
|  | नहीं चाहिए था |
| 1. गर्भधारणा के समय आपने (ए.एन.सी) पंजीकरण किया था ? |  |
|  | हाँ |
|  | ना |
| 1. आप ए.एन.सी पंजीकरण के लिए कहाँ गए थे? |  |
|  | म्युनिसिपल हेल्थ पोस्ट |
|  | नगर पालिका डिलीवरी गृह |
|  | नगर पालिका अस्पताल |
|  | प्राइवेट अस्पताल |
|  | सरकारी अस्पताल |
|  | ट्रस्ट अस्पताल |
|  | फील्ड कैंप |
|  | अलग |
| 1. कौन से महीने मैं गर्भावस्था (ए.एन.सी) पंजीकरण किया था ? |  |
| 1. आपको गर्भावस्था के दौरान सेवा नोंद कार्ड मिला था ?  (उदा मदर चाइल्ड प्रोटेक्शन कार्ड, टीकाकरण कार्ड) |  |
|  | हाँ |
|  | ना |
| 1. क्या आपने कार्ड को देखा या नहीं देखा |  |
|  | कार्ड को देखा |
|  | कार्ड नहीं देखा |
| ए.एन.सी कार्ड का फोटो लीजिए |  |
| 1. गर्भावस्था के दौरान आपने कितनी बार ए.एन.सी के लिए गए ? |  |
| 1. क्या गर्भावस्था के दौरान निचे दिए हुए ए.एन.सी तपास हुए थे? |  |
|  | वजन |
|  | ऊँचाई |
|  | पेट की जाँच |
|  | रक्त चाप (Blood Pressure) |
|  | पेशाब की जाँच |
|  | हीमोग्लोबिन का तपास |
|  | टिटनेस (टीटी/बूस्टर) इंजेक्शन |
|  | कीड़े की दवाई |
|  | अल्ट्रासाउंड स्कैन (sonography) |
|  | कुछ भी नहीं किया था |
| 1. क्या आपका बच्चा मुंबई या मुंबई के बाहर हुआ? |  |
|  | मुंबई |
|  | मुंबई के बाहर |
| 1. आपके बच्चे का जनम घर या अस्पताल में हुआ ? |  |
|  | अस्पताल |
|  | घर |
| 1. आपके बच्चे का जनम कौन से अस्पताल में हुआ ? |  |
|  | नगर पालिका स्वास्थ्य पोस्ट |
|  | नगर पालिका डिलीवरी गृह |
|  | नगर पालिका अस्पताल |
|  | प्राइवेट अस्पताल |
|  | सरकारी अस्पताल |
|  | ट्रस्ट अस्पताल |
|  | अलग |
| 1. आप की डिलीवरी किस प्रकार हो गई? |  |
|  | नोर्मल |
|  | सिझेरियन |
| 1. जन्म के समय वजन लिखिए (जैसे 2500 ,अगर पता नहीं है, तो 9999 लिखिए ) |  |
| 1. डिलीवरी के बाद आप चेक-उप के लिए डॉक्टर से पहली बार कब मिली ? |  |
|  | नहीं मिली |
|  | बच्चे के जन्म के 2 दिनों के बीच |
|  | जन्म देने के बाद 3-7 दिनों के बीच |
|  | जन्म देने के बाद 8-42 दिनों के बीच |
|  | बच्चे के जन्म के बाद बहुत दिनों के लिए अस्पताल में रुके |
| 1. इस अस्पताल जाने का कारन क्या था ? (जो लागु है वो सब बॉक्स में टिक करना) |  |
|  | टीकाकरण |
|  | माँ को तकलीफ थी |
|  | गर्भनिरोधक सलाह |
|  | माता की सामान्य जाँच |
|  | बच्चे की सामान्य जाँच |
|  | बच्चे को तकलीफ थी |
|  | अलग |
| **# Family planning** **अब मैं आपसे परिवार नियोजन के बारे में पूछूंगी** | |
| 1. आपकी पिछली माहवारी कब हुई (LMP)? |  |
|  | पिछले 6 महीने में |
|  | छ्ह महीने से ज्यादा हो गए |
|  | सबसे छोटे बच्चे के जन्म से पहले |
|  | मीनोपॉज या ऑपरेशन |
|  | कभी माहवारी नहीं आयी है |
| 1. क्या आप अभी बच्चा नहीं या देर से होने के लिए कोई तरीका अपना रही है? |  |
|  | हाँ |
|  | ना |
| 1. आपने कौनसा तरीका अपनाया है ? |  |
|  | महिला नसबंदी |
|  | पुरुष नसबंदी |
|  | गोली |
|  | आय.यु. डी /लूप |
|  | गर्भनिरोधक इंजेक्शन |
|  | इम्प्लांट्स |
|  | कंडोम /निरोध |
|  | महिलाओं के लिए कंडोम |
|  | डायाफ्राम |
|  | फोम/जेली |
|  | सुरक्षित काल |
|  | अद्धपतन |
|  | केवल स्तनपान |
| 1. आप को यह कहाँ से मिला ? |  |
|  | नगर पालिका हेल्थ पोस्ट और डिस्पेंसरी |
|  | नगर पालिका मैटरनिटी होम |
|  | नगर पालिका अस्पताल |
|  | प्राइवेट चिकित्सक |
|  | प्राइवेट अस्पताल |
|  | सरकारी अस्पताल |
|  | दवाई की दूकान |
|  | एफ.पी.आय |
|  | एन.जी.ओ |
|  | सी एच व्ही |
|  | अलग |
| 1. क्या आपने पिछले एक साल में इमरजेंसी गर्भधारणा नियमन के तरीको का प्रयोग किया है? |  |
|  | हाँ |
|  | ना |
| 1. आपने कितनी बार इमरजेंसी गर्भधारणा नियमन के तरीको का प्रयोग किया है? |  |
| 1. क्या आप पहले कभी भी बच्चा नहीं या देर से होने के लिए कोई तरीका अपनाया था? |  |
|  | हाँ |
|  | ना |
| 1. आपके बच्चे के जनम के बाद क्या आपको माहवारी आना शुरू हो गया है? |  |
|  | हाँ |
|  | ना |
| 1. मैं अभी आगे के बारे में आप से कुछ सवाल पूछूंगी\| क्या आपको और बच्चे चाहिए या आपको और बच्चे नहीं चाहिए? |  |
|  | और एक बच्चा चाहिए |
|  | कुछ तय नहीं किया है/पता नहीं |
|  | उसने बोला कि वह प्रेगनेंट नहीं हो सकती है |
|  | और बच्चा नहीं चाहिए |
| 1. दुसरे बच्चे के जनम के लिए आप अभी से कितने दिन इंतज़ार करना चाहेंगी? |  |
|  | दो साल से कम इंतज़ार करना है |
|  | दो साल से ज्यादा इंतज़ार करना है |
|  | अलग |
|  | अभी कुछ तय नहीं किया है/पता नहीं |
| 1. आपने बताया कि आपको और बच्चे नहीं चहिये। क्या आप बता सकती हैं कि आप प्रेगनेंसी रोकने के लिए परिवार नियोजन का कोई साधन इस्तेमाल क्यों नहीं कर रही हैं? कोई दूसरा कारण? |  |
|  | विधवा/तलाकशुदा |
|  | प्रजनन क्षमता संबंधी कारण |
|  | शारीरिक सम्बन्ध नहीं है |
|  | शारीरिक सम्बन्ध कभी कभी रखते है |
|  | मीनोपॉज या ऑपरेशन |
|  | प्रेगनेंट नहीं हो सकती है |
|  | सबसे छोटे बच्चे के जनम के बाद से माहवारी नहीं आई है |
|  | स्तन पान |
|  | भगवान पर निर्भर है |
|  | इस्तेमाल करने के पक्ष में नहीं है: |
|  | वह पक्ष में नहीं है |
|  | पति पक्ष में नहीं है |
|  | दुसरे लोग पक्ष में नहीं है |
|  | धर्म में निषेध है |
|  | जानकारी का अभाव: |
|  | किसी साधन के बारे में जानकारी नहीं है |
|  | कहाँ मिलता है यह जानकारी नहीं है |
|  | साधन सम्बन्धी कारण: |
|  | दुस्परिणाम/स्वास्थ्य सम्बंधी चिंता |
|  | मिलने में असुविधा |
|  | बहुत महंगा |
|  | पसंदीदा साधन उपलब्ध नहीं था |
|  | कोई साधन उपलब्ध नहीं था |
|  | इस्तेमाल करने में परेशानी |
|  | शरीर की सामान्य प्रक्रियाओं के साथ हस्तक्षेप |
|  | अलग |
|  | पता नहीं |
| **# IYCF (दो साल या उससे कम उम्र के बच्चे के बारे मैं जानकारी)** | |
| 1. क्या आप ने कभी भी (नाम) को अपना दूध पिलाया है ? |  |
|  | हाँ |
|  | ना |
| 1. जनम के कितनी देर बाद आपने बच्चे को पहली बार अपना दूध पिलाया?  (अगर 1 घंटे से कम है तो 0 लिखिए , 24 घंटे से ज्यादा है तो 88 लिखिए, पता नहीं है तो 99 लिखिए ) |  |
| 1. कितने दिनो के बाद |  |
| 1. जनम के बाद पहले तीन दिनों में, को अपने दूध के अलावा पीने के लिए और कुछ दिया था? |  |
|  | हाँ |
|  | ना |
| 1. जनम के बाद पहले तीन दिनों में को अपने दूध के अलावा पीने के लिए और कुछ दिया था? | |
|  | सिर्फ स्तनपान |
|  | सादापानी |
|  | शक्कर या ग्लूकोज का पानी |
|  | ग्राइपवाटर / बालघुटी |
|  | चीनी, नमक का पानी |
|  | लिक्वीड या फल का रस |
|  | इन्फेंट फार्मूला जैसे लेक्टोजन |
|  | चाय |
|  | शहद |
|  | बालघुटी |
|  | अलग |
| 1. क्या आप बच्चे को अभी भी अपना दूध पिला रहे है? |  |
|  | हाँ |
|  | ना |
| 1. कितने महीने स्तनपान को किया गया था ? |  |
| 1. रात को सूरज डूबने और आज सबेरे सूरज निकलने के बीच आपने को कितनी बार अपना दूध पिलाया ? |  |
| 1. कल दिन में कितनी बार आपने को अपना दूध पिलाया था ? |  |
| 1. क्या बच्चे को कल या कल रात में निप्पल वाली बोतल से दूध पिलाया? |  |
|  | हाँ |
|  | ना |
| 1. मैं आप से कुछ तरल पदार्थ के बारे में पूछूंगी जो शायद आप ने (नाम) को कल दिन या रात में दिया होगा. क्या ने इनमे से कुछ पीया? | |
|  | पानी |
|  | हाँ |
|  | ना |
|  | पता नहीं |
|  | इन्फेंट फार्मूला जैसे लेक्टोजन |
|  | हाँ |
|  | ना |
|  | पता नहीं |
|  | दूध जैसे टिन, पाउडर या किसी जानवर का दूध |
|  | हाँ |
|  | ना |
|  | पता नहीं |
|  | लस्सी, छास या दही का शरबत |
|  | हाँ |
|  | ना |
|  | पता नहीं |
| 1. कल दिन या रात में (नाम) ने कितनी बार किसी भी तरह का दूध पीया ? (अगर 7 या 7 से ज्यादा है तो,7 लिखिए ) |  |
|  | फल का रस |
|  | हाँ |
|  | ना |
|  | पता नहीं |
|  | पतला सूप |
|  | हाँ |
|  | ना |
|  | पता नहीं |
|  | चाय या कॉफ़ी |
|  | हाँ |
|  | ना |
|  | पता नहीं |
|  | कोल्ड ड्रिंक्स |
|  | हाँ |
|  | ना |
|  | पता नहीं |
| 1. दूसरा कोई तरल पदार्थ |  |
|  | हाँ |
|  | ना |
|  | पता नहीं |
| 1. क्या आप ने को कभी किसी तरह का ठोस या नरम चीज़ें खाने के लिए दिया है ? |  |
|  | हाँ |
|  | ना |
|  | पता नहीं |
| 1. अभी मैं आपसे कुछ खाने के बारे में पुछुगी जो शायद आप ने (नाम ) को कल दिन या रात में दिया होगा\| क्या ने इनमे से कुछ खाया ? | |
|  | बेबी फ़ूड |
|  | हाँ |
|  | ना |
|  | पता नहीं |
| 1. खिचड़ी, हलवा , रोटी, चपाती, चावल, इडली, या किसी भी अन्य खाद्य पदार्थ अनाज से बना | |
|  | हाँ |
|  | ना |
|  | पता नहीं |
| 1. लौकी गाजर शकार्खंड (रतालू ) जो अन्दर से पीला या नारंगी रंग का है | |
|  | हाँ |
|  | ना |
|  | पता नहीं |
| 1. आलू ,सुरंद , अरबी, या दूसरी कोई चीज़ जो खंड से बनी हो | |
|  | हाँ |
|  | ना |
|  | पता नहीं |
| 1. गहरे हरे रंग के पत्ते की सब्जी | |
|  | हाँ |
|  | ना |
|  | पता नहीं |
| 1. पके हुए आम , पपीता, खरबूजा या कटहल | |
|  | हाँ |
|  | ना |
|  | पता नहीं |
| 1. दूसरा कोई फल या सब्जी |  |
|  | हाँ |
|  | ना |
|  | पता नहीं |
| 1. कलेजी, गुर्दा , भेजा जैसा मांस |  |
|  | हाँ |
|  | ना |
|  | पता नहीं |
| 1. मुर्गा, बतख या कोई और चिड़िया का मांस |  |
|  | हाँ |
|  | ना |
|  | पता नहीं |
| 1. किसी और चीज़ का मांस |  |
|  | हाँ |
|  | ना |
|  | पता नहीं |
| 1. अंडे |  |
|  | हाँ |
|  | ना |
|  | पता नहीं |
| 1. ताज़ा या सुखा मछली |  |
|  | हाँ |
|  | ना |
|  | पता नहीं |
| 1. मटर , मसूर जैसे चीज़ से बना |  |
|  | हाँ |
|  | ना |
|  | पता नहीं |
| 1. बादाम जैसी चीज़ |  |
|  | हाँ |
|  | ना |
|  | पता नहीं |
| 1. पनीर दही या कोई दूध से बनी चीज़ |  |
|  | हाँ |
|  | ना |
|  | पता नहीं |
| 1. तेल घी या मक्खन से बना खाना |  |
|  | हाँ |
|  | ना |
|  | पता नहीं |
| 1. मीठी चीजे जैसे चॉकलेट, मिठाई, कैंडी, पेस्ट्री, केक या बिस्किट,आइस क्रीम |  |
|  | हाँ |
|  | ना |
|  | पता नहीं |
| 1. नल्ली या वेफर्स |  |
|  | हाँ |
|  | ना |
|  | पता नहीं |
| 1. वड़ा , समोसा , भजिया जैसी ताली हुई चीज़े |  |
|  | हाँ |
|  | ना |
|  | पता नहीं |
| 1. नूडल्स |  |
|  | हाँ |
|  | ना |
|  | पता नहीं |
| 1. चायनीज़ भेल / पकोडा |  |
|  | हाँ |
|  | ना |
|  | पता नहीं |
| 1. ब्रेड / पाव/ टोस्ट / बटर / खारी/पानीपूरी |  |
|  | हाँ |
|  | ना |
|  | पता नहीं |
| 1. कोई अन्य ठोस या नरम खाना |  |
|  | हाँ |
|  | ना |
|  | पता नहीं |
| 1. कल दिन और रात में ने कितनी बार तरल पदार्थ के अलावा ठोस या नरम खाना खाया ? |  |
|  |  |
| **टीकाकरण** | |
| 1. क्या बच्चे का टीकाकरण कार्ड है? (अगर टीकाकरण कार्ड है तो उससे देख कर लिखिए) |  |
|  | हाँ |
|  | ना |
| 1. क्या बच्चे का टीकाकरण कार्ड है? (अगर टीकाकरण कार्ड है तो उससे देख कर लिखिए) |  |
|  | कार्ड को देखा |
|  | कार्ड नहीं देखा |
| कार्ड का फोटो लीजिए |  |
| 1. बी. सी.जी (जनम के समय) |  |
|  | हाँ |
|  | ना |
|  | पता नहीं |
| 1. पोलिओ 0 (जनम के समय ) |  |
|  | हाँ |
|  | ना |
|  | पता नहीं |
| 1. हेपेटाइटिस (बी-0) (जनम के समय ) |  |
|  | हाँ |
|  | ना |
|  | पता नहीं |
| 1. पोलिओ -1 (ढेड़ महीने) |  |
|  | हाँ |
|  | ना |
|  | पता नहीं |
| 1. डी.पी.टी -1 (ढेड़ महीने) |  |
|  | हाँ |
|  | ना |
|  | पता नहीं |
| 1. हेपेटाइटिस (बी-1) (ढेड़ महीने) |  |
|  | हाँ |
|  | ना |
|  | पता नहीं |
| 1. आय.पी.वी -1 (ढेड़ महीने) |  |
|  | हाँ |
|  | ना |
|  | पता नहीं |
| 1. पेंटा - 1 (ढेड़ महीने) |  |
|  | हाँ |
|  | ना |
|  | पता नहीं |
| 1. पोलिओ - 2 (ढाई महीने) |  |
|  | हाँ |
|  | ना |
|  | पता नहीं |
| 1. डी.पी.टी - 2 (ढाई महीने) |  |
|  | हाँ |
|  | ना |
|  | पता नहीं |
| 1. हेपेटाइटिस (बी-2) (ढाई महीने) |  |
|  | हाँ |
|  | ना |
|  | पता नहीं |
| 1. पेंटा - 2 (ढाई महीने) |  |
|  | हाँ |
|  | ना |
|  | पता नहीं |
| 1. पोलिओ - 3 (साड़े तीन महीने) |  |
|  | हाँ |
|  | ना |
|  | पता नहीं |
| 1. डी.पी.टी - 3 (साड़े तीन महीने) |  |
|  | हाँ |
|  | ना |
|  | पता नहीं |
| 1. हेपेटाइटिस (बी-3) (साड़े तीन महीने) |  |
|  | हाँ |
|  | ना |
|  | पता नहीं |
| 1. आय.पी.वी - 2 (साड़े तीन महीने) |  |
|  | हाँ |
|  | ना |
|  | पता नहीं |
| 1. पेंटा - 3 (साड़े तीन महीने) |  |
|  | हाँ |
|  | ना |
|  | पता नहीं |
| 1. मिझलस (9 महीने के बाद) |  |
|  | हाँ |
|  | ना |
|  | पता नहीं |
| 1. एम.एम.आर (ढेड़ साल) |  |
|  | हाँ |
|  | ना |
|  | पता नहीं |
| 1. ज्यादातर टिकाकरण कहाँसे लगवाया |  |
|  | नगर पालिका हेल्थ पोस्ट |
|  | नगर पालिका अस्पताल |
|  | प्राइवेट जी.पी |
|  | प्राइवेट अस्पताल |
|  | सरकारी अस्पताल |
|  | अर्बन हेल्थ सेंटर |
|  | आउटरीच कैंप |
| **बच्चे की बीमारी और उसका इलाज** | |
| 1. क्या "बच्चे का नाम" को पिछले दो सप्ताह (15 दिन ) में कभी जुलाब (पतला संडास) हुआ था ? |  |
|  | हाँ |
|  | ना |
| 1. जुलाब (पतला संडास ) के लिए क्या आपने कही से सलाह लिया या इलाज़ करवाया ? |  |
|  | हाँ |
|  | ना |
| 1. क्या "बच्चे का नाम" को पिछले दो सप्ताह (15 दिन ) में कभी बुखार हुआ था ? |  |
|  | हाँ |
|  | ना |
| 1. क्या "बच्चे का नाम" को पिछले दो सप्ताह (15 दिन ) में कभी खासी हुई ? |  |
|  | हाँ |
|  | ना |
| 1. जब "बच्चे का नाम" को खासी थी तो क्या वह छोटी छोटी सामान्य से तेज साँसे लेता था /थी या उसे साँस लेने में परेशानी हो रही थी ? |  |
|  | हाँ |
|  | ना |
| 1. बीमारी के दौरान क्या सीने में तकलीफ थी नाक बंद या बह रही थी ? |  |
|  | केवल छाती |
|  | केवल नाक |
|  | दोनों |
|  | पता नहीं |
| 1. बुखार या सीने में तकलीफ के लिए क्या आपने कही से सलाह लिया या इलाज़ करवाया ? |  |
|  | हाँ |
|  | ना |
| 1. पिछले दो सप्ताह मैं को कोई भी अन्य बीमारी थी? |  |
|  | हाँ |
|  | ना |
| 1. बीमारी किस तरह की थी ? |  |
|  | शरीरपर छोटे छोटे दाने |
|  | उल्टी |
|  | त्वचा का इन्फेक्शन |
|  | कान का इन्फेक्शन |
|  | पीलिया |
|  | पेट में समस्या |
|  | पेशाब में समस्या |
|  | फिट |
|  | चोट |
|  | अन्य |
| **इंटरवेंशन प्रोसेस** |  |
| 1. क्या आप अपने प्लाट में स्नेहा के बारे में जानते हैं? |  |
|  | हाँ |
|  | ना |
| 1. स्नेहा कौन से विषय काम कर रहे है इसकी आपको जानकारी है? |  |
|  | हाँ |
|  | ना |
| 1. क्या-क्या जानकारी दी जाती हैं? |  |
|  | परिवार नियोजन |
|  | टीकाकरण |
|  | वजन और ऊंचाई नापा |
|  | पोषण और स्वास्थ्य शिक्षा |
|  | हिंसा पीड़ितों के लिए काउंसलिंग |
|  | स्वास्थ्य सुविधा मिलने के लिए सहयाता की |
|  | रेफरल सेवाएं |
|  | अलग |
| 1. पिछले एक साल में आपको या आपके परिवार को स्नेहा सेंटर से क्या कोई सुविधा मिली |  |
|  | हाँ |
|  | ना |
| 1. कौनसी सुविधा मिली? |  |
|  | टीकाकरण |
|  | परिवार नियोजन |
|  | वजन और ऊंचाई नापा |
|  | पोषण और स्वास्थ्य शिक्षा |
|  | हिंसा पीड़ितों के लिए काउंसलिंग |
|  | स्वास्थ्य सुविधा मिलने के लिए सहयाता की |
|  | रेफरल सेवाएं |
|  | अलग |
| 1. क्या पिछले एक साल में आप या आप के परिवार वाले कभी स्नेहा सेंटर में गए? |  |
|  | हाँ |
|  | ना |
| 1. आप या आप के परिवार वाले वहां क्यों गए? |  |
|  | वजन और ऊंचाई नापने के लिए |
|  | ग्रुप मीटिंग्स |
|  | समारोह |
|  | एनीमिया कैंप |
|  | हिंसा केस पंजीकरण |
|  | टीकाकरण शिबिर |
|  | अलग |
|  | सी ए जी मीटिंग्स |
| 1. क्या पिछले एक साल में स्नेहा सेंटर से किसी ने आपको या आपके परिवार वालों को भेंट दी है? |  |
|  | हाँ |
|  | ना |
| 1. वह कितनी बार आपको भेंट देती है? |  |
|  | हर हफ्ते में |
|  | हर15 दिनों में |
|  | हर महीने |
|  | हर दो महीने में |
|  | कभी कभी |
|  | कभी नहीँ |
| **(पिछले एक साल ) स्नेहा सेंटर के कार्यक्रम बारे में जानकारी** | |
| 1. पिछले एक साल में क्या आप या आपके परिवार वालों ने स्नेहा सेंटर के किसी कार्यक्रम में हिस्सा लिया? |  |
|  | हाँ |
|  | ना |
| 1. आप या आपके परिवार वालों ने स्नेहा सेंटर के कौन से कार्यक्रम में हिस्सा लिया? |  |
|  | ग्रुप मीटिंग्स |
|  | माता-पिता की मीटिंग्स |
|  | पुरुषों की मीटिंग्स |
|  | समारोह |
|  | एनीमिया कैंप |
|  | गैर सरकारी संगठनों / स्वास्थ्य केन्द्रों कि भेंट |
|  | अलग |
|  | आप ने कौन से कार्यक्रम में हिस्सा लिया? |
|  | गोद भराई |
|  | उष्टावन |
|  | कपल एक्टिविटी |
|  | मंथली व्हायलन्स कैंपेन |
|  | खाना-खजाना |
|  | अलग |
|  | स्तनपान सप्ताह |
|  | पोषण सप्ताह |
|  | सी ए जी क्षमता निर्माण |
|  | 16 दिनों सक्रियता |
| 1. क्या यह जानकारी आप के लिए फायदेमंद थी? |  |
|  | हाँ |
|  | ना |
|  | पता नहीं |
| 1. क्या आप अपने बस्ती के ऐसे किसी को जानते हैं जो स्नेहा के साथ स्वयंसेवी के तौर पर जुड़े हैं? |  |
|  | हाँ |
|  | ना |
|  | मैं स्वयंसेवक (Volunteer) हूँ |
|  | पता नहीं |
| 1. पिछले एक साल में क्या स्नेहा के स्वयंसेवक ने कभी आप को भेंट दी? |  |
|  | हाँ |
|  | ना |
| **# आय.सी.डी.एस / बी.एम.सी सर्विस** | |
| 1. पिछले एक साल के दौरान आप या आपके बच्चों को आंगनवाड़ी / आईसीडीएस से कोई सुविधा प्राप्त हुई थी ? |  |
|  | हाँ |
|  | ना |
|  | पूरक पोषण/ आहार |
|  | 6 महीने से 6 साल तक के आपके बच्चे |
|  | आपके गर्भावस्था के समय |
|  | स्तनपान करवाने के समय |
|  | कुछ भी नहीं |
|  | वज़न /ऊँचाई नापा |
|  | 6 महीने से 6 साल तक के आपके बच्चे |
|  | आपके गर्भावस्था के समय |
|  | स्तनपान करवाने के समय |
|  | कुछ भी नहीं |
|  | स्वास्थ्य और पोषण शिक्षा |
|  | आपके गर्भावस्था के समय |
|  | स्तनपान करवाने के समय |
|  | स्कूल पूर्व शिक्षा |
|  | कुछ भी नहीं |
| 1. पिछले एक साल के दौरान आप या आपके बच्चों को आंगनवाड़ी/आईसीडीएस से कोई सुविधा के लिए रेफरल किया था? |  |
|  | हाँ |
|  | ना |
| 1. पिछले एक साल के दौरान आप या आपके बच्चों को बी.एम.सी से से कोई सुविधा प्राप्त हुई थी ? |  |
|  | हाँ |
|  | ना |
| 1. पिछले एक साल के दौरान, आपको बी.एम.सी से कोई सुविधा प्राप्त हुई थी ? अगर हाँ है तो कौन सी सुविधाए आपको मिली? |  |
|  | प्रसव पूर्व देखभाल |
|  | प्रसव देखभाल |
|  | प्रसव के बाद देखभाल (PNC) |
|  | परिवार नियोजन |
|  | माँ की बीमारी के लिए |
|  | स्वास्थ्य कार्यकर्ता ने भेट दी |
|  | अलग |
|  | कुछ भी नहीं |
|  | कुपोषण |
|  | टीकाकरण |
|  | कीड़े की दवाई |
|  | हेल्थ कैंप |
|  | बच्चे की बीमारी के लिए |
| 1. पिछले 1 साल में आपको या आपके परिवार को स्नेहा के अलावा किसी ओर एन.जी.ओ से क्या कोई सुविधा मिली ? |  |
|  | हाँ |
|  | ना |
| 1. कौनसा एन.जी.ओ? |  |
|  | एफ एच एफ |
|  | एफपीआय |
|  | पार्थ |
|  | अरमान |
|  | नासो |
|  | अपनालय |
|  | अमेरि केयर्स |
|  | Vision Rescue |
|  | धार्मिक ट्रस्ट |
|  | सत्य साई ट्रस्ट |
|  | डॉक्टर्स फॉर यू |
|  | रिलायंस फाउंडेशन |
|  | निरामया फाउंडेशन |
|  | स्त्री मुक्ति संगठना |
|  | अलग |
|  | अन्य एन.जी.ओ का नाम लिखिए |
| 1. अन्य एनजीओ का नाम |  |
| 1. यह इंटरव्यू के दौरान पी.ओ मौजूद थे? |  |
|  | नहीं |
|  | aaaa |
|  | bbbb |
| जानकारी देनेवाले को बताइये :समय निकाल कर जानकारी देने के लिए धन्यवाद. क्या आप हम से कुछ पूछना चाहते है? |  |
